# Refinement of the androgen response element based on ChIP-Seq in androgen-insensitive and androgen-responsive prostate cancer cell lines

**DOI:** 10.1038/srep32611

**Published:** 2016-09-14

**Authors:** Stephen Wilson, Jianfei Qi, Fabian V. Filipp

**Affiliations:** 1 Systems Biology and Cancer Metabolism, Program for Quantitative Systems Biology, University of California Merced, 2500 North Lake Road, Merced, CA 95343, USA; 2 Marlene and Stewart Greenebaum Cancer Center, Department of Biochemistry and Molecular Biology, University of Maryland School of Medicine, 655 West Baltimore Street, Baltimore MD 21201, USA

## Abstract

Sequence motifs are short, recurring patterns in DNA that can mediate sequence-specific binding for proteins such as transcription factors or DNA modifying enzymes. The androgen response element (ARE) is a palindromic, dihexameric motif present in promoters or enhancers of genes targeted by the androgen receptor (AR). Using chromatin immunoprecipitation sequencing (ChIP-Seq) we refined AR-binding and AREs at a genome-scale in androgen-insensitive and androgen-responsive prostate cancer cell lines. Model-based searches identified more than 120,000 ChIP-Seq motifs allowing for expansion and refinement of the ARE. We classified AREs according to their degeneracy and their transcriptional involvement. Additionally, we quantified ARE utilization in response to somatic copy number amplifications, AR splice-variants, and steroid treatment. Although imperfect AREs make up 99.9% of the motifs, the degree of degeneracy correlates negatively with validated transcriptional outcome. Weaker AREs, particularly ARE half sites, benefit from neighboring motifs or cooperating transcription factors in regulating gene expression. Taken together, ARE full sites generate a reliable transcriptional outcome in AR positive cells, despite their low genome-wide abundance. In contrast, the transcriptional influence of ARE half sites can be modulated by cooperating factors.

Prostate cancer is the most common malignancy in American men^1^,^2^. Although localized prostate cancer is curable by surgery, the primary treatment for metastatic prostate cancer remains androgen deprivation therapy (ADT). However, the success of ADT is not guaranteed. Despite an initial response, advanced prostate cancer develops almost invariably resistance to ADT and progresses to a lethal disease stage called castration-resistant prostate cancer (CRPC). Thus, understanding the mechanisms underlying the development of CRPC is of critical importance for basic and clinical research.

Reactivation of the androgen receptor (AR, GeneBank: 367) under low androgen condition is believed to drive the development of CRPC^3^,^4^. AR belongs to the nuclear receptor superfamily and plays an important role in the physiology of normal prostate gland and progression of prostate adenocarcinoma (PRAD). The AR gene is located on the X chromosome at Xq11-12 and contains eight exons encoding a 919 amino acid-long protein. The AR protein consists of an N-terminal transactivation domain (NTD), a central DNA binding domain (DBD), a hinge region, and a C-terminal ligand-binding domain (LBD)^5^. Unliganded AR is sequestered in the cytoplasm by a chaperone complex. Upon ligand binding to the LBD, AR changes its conformation, dissociates from the chaperone complex, dimerizes, and translocates into the nucleus. Once translocated into the nucleus the AR dimer binds to the androgen response elements (AREs) present in promoter or enhancer elements of its target genes, and recruits co-activators or co-repressors to regulate gene expression.

Reactivation of the AR in CRPC can be due to changes of the AR or its steroid ligands^6^,^7^,^8^,^9^,^10^,^11^,^12^. Known alterations of the AR include somatic gene amplification and/or over-expression that increase AR mediated response to low androgen levels, AR mutations that change ligand specificity to allow for activation by other steroids, and generation of AR splice variants (AR-Vs) that lack the LBD and are constitutively active even in the absence of androgen. CRPC cells can also synthesize androgens themselves by conversion of testosterone derivatives or *de novo* by biosynthesis from cholesterol. Such intra-tumoral androgen synthesis permits maintenance of certain intracellular androgen levels and results in reconquered mitogenic AR activity^13^,^14^,^15^.

Studies of AR chromatin binding by chromatin immunoprecipitation (ChIP) approaches like ChIP-on-chip or ChIP in combination with next generation sequencing (ChIP-Seq) have shed light onto the mechanisms of global regulation of AR activities in prostate cancer cell lines or tissues. Most AR chromatin binding studies were performed in the LNCaP cell line or its sublines^3^,^4^,^6^,^7^,^9^,^10^,^11^,^12^, which are highly sensitive and responsive to androgen stimulation. LNCaP or its sublines express a full-length AR with point mutation of T877A at the LBD^16^. In contrast, there are limited studies on global AR binding in the CWR22Rv1 cell line^4^,^8^, another AR-positive but androgen-insensitive PRAD cell line. The CWR22Rv1 prostate cancer cell line expresses AR full-length (AR(FL)) with a duplicated DBD in exon 3^17^,^18^,^19^ and an AR splice variant, AR(V), lacking a LBD, thus becoming constitutively active^4^,^8^,^11^,^20^. In contrast to the LNCaP cell line where the AR depends on androgen activation, the CWR22Rv1 cell line shows constitutively active AR with limited changes in expression of AR target genes in the presence or absence of androgens^19^,^21^.

AREs are well studied but poorly defined and have been shown to contain two hexamers with a three base-pair spacer with an inverted repeat in the second hexamer^22^. The sequence elements similar to this canonical ARE have been identified in some ChIP-Seq studies, whereas half AREs or tandem repeats of two hexamers were also found in other ChIP-Seq or ChIP-on-chip studies. In the past, studies revealed binding motifs adjacent to the AR binding sites but belonging to other transcription factor families such as the forkhead box A1 protein (FOXA1, GeneBank: 3169). Cooperative interactions facilitate chromatin binding of the AR and contribute to a promiscuous behavior of AREs^23^,^24^,^25^. AREs and adjacent transcription binding motifs have been well described in LNCaP cells but remain to be defined in CWR22Rv1 cells. Therefore, the purpose of our AR ChIP-Seq study is to further characterize the ARE and identify cooperation with adjacent transcription binding motifs in androgen-responsive and androgen-insensitive prostate cancer cell lines.

## Methods

### Cell culture

CWR22Rv1 is a human prostate carcinoma epithelial cell line derived from a xenograft that was serially propagated in mice after castration-induced regression and relapse of the parental, androgen-dependent CWR22 xenograft^26^,^27^ (CRL-2505, American Type Culture Collection, Manassas, VA). The CWR22Rv1 prostate cancer cell line was kindly provided by Dr. James Jacobberger (Case Western Reserve University, Cleveland, OH), and are maintained in RPMI 1640 medium supplemented with 10% FBS and antibiotics. Cells are regularly tested to ensure that they are mycoplasma-free. All experimental protocols were approved by the Institutional Review Board at the University of California Merced. The study was carried out as part of IRB UCM13-0025 of the University of California Merced and as part of dbGap ID 5094 on somatic mutations in cancer and conducted in accordance with the Helsinki Declaration of 1975.

### Knockdown of AR with shRNA

Lentiviral vectors encoding AR shRNA were purchased from Open Biosystems, and packaged in 293T cells by the calcium phosphate transfection. The supernatant containing lentiviral particles were collected 48 hours after transfection. CWR22Rv1 cells were transduced with the supernatant of lentiviral particles in the presence of polybrene (8 μg/ml) for 24 hours before replacement with the fresh growth media. Cells were analyzed at 72 hours post-transduction. The knockdown efficiency was confirmed by quantitative real time polymerase chain reaction (qRT-PCR) and Western-blot analysis (Supplementary Figure 1).

### qRT-PCR analysis

Total RNA from prostate cancer cells was extracted using a mammalian RNA mini preparation kit (Sigma, GenElute, RTN10, Darmstadt, Germany) and then digested with deoxyribonuclease I (Sigma, AMPD1, Darmstadt, Germany). Complementary DNA (cDNA) was synthesized using random hexamers. Triple replicate samples were subjected to SYBR green (SYBR green master mix, Qiagen SABiosciences) qRT-PCR analysis in an Eco system (Illumina, San Diego). Gene expression profiles were analyzed using the ΔΔCT method. RT QPCR threshold cycle (CT) values were normalize the housekeeping gene cyclophilin A (PPIA, peptidylprolyl isomerase A, GeneBank: 5478). The following primers served for qRT-PCR analysis of human gene transcripts: PPIA: 5′-GACCCAACACAAATGGTTC-3′; 5′-AGTCAGCAATGGTGATCTTC-3′; AR: 5′-CTCCGCTGACCTTAAAGACATC-3′; 5′-TGCCCCCTAAGTAATTGTCCTT-3′.

### Western-blot analysis

Whole cell lysates were harvested using radio-immunoprecipitation assay (RIPA) buffer composed of 50 mM trisaminomethane hydrochloride (Tris-HCl) pH 7.5, 150 mM sodium chloride (NaCl), 1% Triton X-100, 0.1% sodium dodecyl sulfate (SDS), 0.1% sodium deoxycholate, 1.0 mM EDTA, 1.0 mM sodium orthovanadate, and 1x protease inhibitor cocktail. Lysates were subjected to sodium dodecyl sulfate-polyacrylamide gel electrophoresis (SDS-PAGE) and proteins transferred to a nitrocellulose membrane (GE Healthcare Life Sciences). The membrane was probed with an AR antibody (Sigma EMD Millipore, PG21, 06-680, Darmstadt, Germany) or actin antibody (Sigma EMD Millipore, A2066, Darmstadt, Germany) followed by a secondary antibody conjugated to fluorescent dye, and blots were imaged using the odyssey detecting system (LI-COR Biotechnology).

### Chromatin immunoprecipitation

Cells were crosslinked using 1% formaldehyde for 10 min at 298 K. Formaldehyde was diluted to a final concentration of 125 mM by adding 5 M glycine. Nuclear extracts were collected and sonicated to obtain 300 bp chromatin fragments using the Covaris S2 ultrasonicator (Covaris, Woburn, MA). 100 μg of chromatin was incubated with 5.0 μg of AR antibodies (Sigma EMD Millipore, PG21, 06–680, Darmstadt, Germany) overnight at 277 K followed by incubation with 30 μl of protein A/G beads for 4 hours. After four washes, crosslinking was reversed, and chromatin was digested with ribonuclease A (RNaseA) followed by proteinase K. The DNA was purified using spin columns. The size of the DNA was confirmed by a bioanalyzer (Agilent Biotechnologies, Savage, MD).

### Next generation sequencing and ChIP-seq data analysis

The purified DNA library was sequenced using an Illumina HighSeq2000 at the Sanford-Burnham Medical Research Institute, National Genome Library Core Facility (Lake Nona, FL). This study included next generation sequencing reads of ChIP-Seq experiments of the androgen-independent CWR22Rv1 cell line as well as of the androgen-dependent LNCaP cell line. For the CWR22Rv1 cell line^27^ we acquired total AR binding by ChIP-Seq. In addition, we compared the data to AR splice variant-specific isoforms AR(FL) and AR(V) as described^28^. For the LNCaP cell line^29^ we assessed conditions of testosterone and ethanol treatment as described^30^. Sequenced regions were aligned to the reference human genome 19 using the Bowtie alignment program that utilizes an extended Burrows-Wheeler indexing for an ultrafast memory efficient alignment^31^. Peak calling utilized a model-based analysis of ChIP-Seq (MACS) algorithm^32^,^33^. The overlap analysis, plot of genomic location, sequence extraction, motif identification, and peak filtering were performed using ChIPseek: a web-based analysis for ChIP data^34^. ChIPseek also employs scripts from BEDtools^35^ using a genome binning algorithm used by the UCSC genome browser to sort genomic regions into groups along the length of chromosome^36^. Data visualization was carried out using the integrative genomics viewer^37^. The tool genomic identification of significant targets in cancer (GISTIC), 2.0.21^38^, was used to identify genomic regions that are significantly gained or lost across a set of paired normal and tumors samples of 492 specimen on Agilent SNP 6.0 gene expression microarrays G4502A_07_01. Arm-level amplification of HighSeq2000 data was estimated and compared to near diploid averages^39^. Events whose length was greater or less than 50% of the chromosome arm on which they resided were called arm-level or focal events, respectively. Segmented level 3 tumor copy number data relative to normal samples was used as input for GISTIC 2.0.21 and aligned to HG19. For significant loci and genes a cutoff q-value of 0.05 was applied.

### Motif analysis based on position site specific matrix models

Computational response element searching algorithms are able to estimate a sequence’s likelihood in belonging to the response element of the query transcription factor using position site specific matrices where each position in the query transcription factor model gives each of the four letters in the DNA alphabet a score based on the probability of that nucleotide being found at that position (Supplementary Table S1)^40^. ChIP-Seq derived ARE motif logos are deposited in transcription factor databases. Experimental transcription factor matrices based on 42956 and 79065 ChIP-Seq AR-binding events for ARE full and half sites, respectively, are referenced under accession M08907 and M08908 in TRANSFAC 2016.3, accession AR in Jaspar, and accession ANDR_HUMAN in HOCOMOCO. Summation into a logs-odd score is converted into a p-value assuming a zero-order background model, and all response elements less than the threshold are reported^41^. Motif discovery, motif enrichment, and motif scanning used the multiple expectation maximization for motif elicitation (MEME) and discriminative regular expression motif elicitation (DREME) suite software toolkits from a set of user supplied unaligned sequences for ChIP-Seq regions^42^. *De novo* motif analysis programs MEME and DREME identifies similar reoccurring DNA sequences, and allows easy submission genomic sequence databases to find similarity to previously studied DNA binding protein motifs^43^,^44^. After a motif of interest is discovered the genomic sequences of the ChIP sequenced data is scanned using the MEME suite software find individual motif occurrences (FIMO)^41^ for individual motif occurrences using a position specific matrix to compute a log-likelihood ratio score for each submitted sequence. The position specific matrix is used further to analyze the sequenced data for motif enrichment for identifying potential co-activators within the data^45^. Transcription factor complexes were inferred from ChIP-Seq data using spaced motif analysis (SPAMO)^46^.

### Microarray analysis

CWR22v1 cells were transduced with lentiviral pLKO.1 control vector or AR shRNA for 72 h. Total RNA was isolated from cells, and 500 ng was used for synthesis of biotin-labeled cRNA using an RNA amplification kit (Ambion, Thermo Fisher Scientific, Waltham, MA). Biotinylated cRNA was labeled by incubation with streptavidin-Cy3 to generate a probe for hybridization with the GeneChip Human Transcriptome Array 2.0 (Affymetrix Inc, Santa Clara, CA). Four samples from two experimental groups (n = 2 per group) were hybridized to the chip to obtain raw gene expression data, which was processed to obtain raw data in the form of expression intensities. Raw data was then exported for further processing and analysis using R statistical software version 2.15 in combination with the BioConductor package^47^. The raw signal intensities were background corrected by using array-specific measures of background intensity based on negative control probes, prior to transformation and normalization using the variance stabilization (VSN)^48^. The dataset was then filtered to remove probes not detected (detection score < 0.95) in any sample. Differential expression between experimental groups was assessed by generating relevant contrasts corresponding to the two-group comparison and was evaluated using the linear models for microarray analysis (LIMMA) package^47^. Raw p-values were corrected for multiple testing using the false discovery rate controlling procedure of Benjamini and Hochberg, and adjusted p-values below 0.05 were considered significant^49^. Significant probe lists were then annotated using the relevant annotation file (HumanHT-12_V4_0_R1_15002873_B).

## Results

### Identification of ARE motifs based on ChIP-Seq pattern, models, or database knowledge

**Figure 1:**
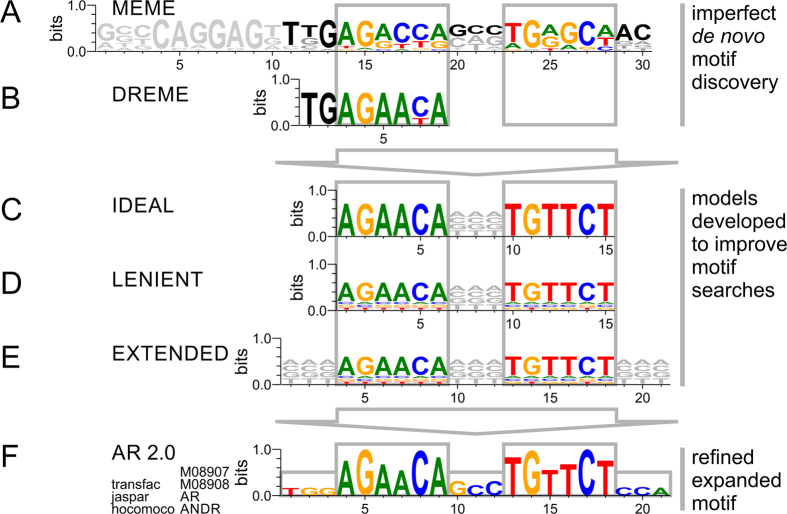
Identification of transcriptional motifs of the androgen receptor based on ChIP-Seq pattern, database knowledge, and position site specific matrix models. (**A**) Motif discovery based on fixed-length patterns or (**B**) short sequence pattern in conjunction with motif databases result in sparse, imperfect motifs. Model-based searches starting from (**C**) ideal model, (**D**) lenient model, or (**E**) extended model provides exhaustive description of motif space in ChIP-Seq experiment. (**F**) Using the identified AREs within our experiment a refined extended canonical ARE model is proposed and deposited in transcription factor databases under accession numbers M08907 (ARE full site), M08908 (ARE half site), AR, and ANDR.

ChIP-Seq data represents an enrichment of loci related to the binding of the protein of interest selected by the immunoprecipitation. Our ChIP-Seq dataset contained 35073 broad peaks including 4731261 wiggle signals detected by MACS and significantly enriched above the genomic control. 3017 (8.6%) of the detected peaks contained AREs, with many peaks showing multiple motif incidences. Furthermore, we detected DNA motifs resembling ARE sequences in about every 40^th^ wiggle signal. Without putting any knowledge into the search for ARE motifs, we first attempted to conduct a pattern search independent of existing databases. A *de novo* motif discovery search showed an ungapped 30mer logo on the ChIP-Seq data using the MEME tool^50^ (Fig. 1A). The 30mer contained not only a palindromic ARE-full site but also information on adjacent bases in the proximity of the ARE. Motif searches relied on existing database entries matching the ChIP-Seq data to a perfect but shortened ARE half site using the DREME tool^44^ (Fig. 1B). Next, we attempted to identify ARE locations within our ChIP-Seq data using the FIMO tool^41^ with a position site specific matrix as it is defined in motif databases (Supplementary Table S1). This proved problematic as the ARE identified by currently available database models (e.g. Jaspar model MA0007.2)^51^ did not resemble the palindromic ARE sequence described in the literature and were shifted in frame^22^,^52^,^53^,^54^. In order to identify and describe AREs within our genome-wide ChIP-Seq dataset we implemented position site specific matrix (PSSM) models. The initial scan was based on a strict ideal 15mer motif search pattern of two hexamers with a 3mer spacer AGAACANNNTGTTCT (Fig. 1C). However, transcription factor binding is more lenient in its pattern recognition. A lenient 15mer motif search pattern not only dramatically improved detection of ARE occurrences but also allowed for classification of AREs with respect to deviation from a perfect motif (Fig. 1D). The lenient model is distinct from the ideal model such that it allowed for limited mismatch at individual base positions with the core hexamers of full site AREs as frequently observed in binding experiments. In an attempt to elucidate the pattern of the regions surrounding the ARE the lenient motif search pattern was expanded (Fig. 1E). In addition, a half site specific PSSM comprised of an isolated hexamer with flanking sequences captured all ARE motifs, which were lacking a complementary palindromic half site hexamer. Finally we refined and expanded the ARE motif discovered based on our experimental ChIP-Seq data referencing 42956 and 79065 events for ARE full and half site, respectively (Fig. 1F) (Supplementary Table S1).

### Identification and base-specific classification of AREs into 5 tiers utilizing ChIP-Seq data

**Figure 2:**
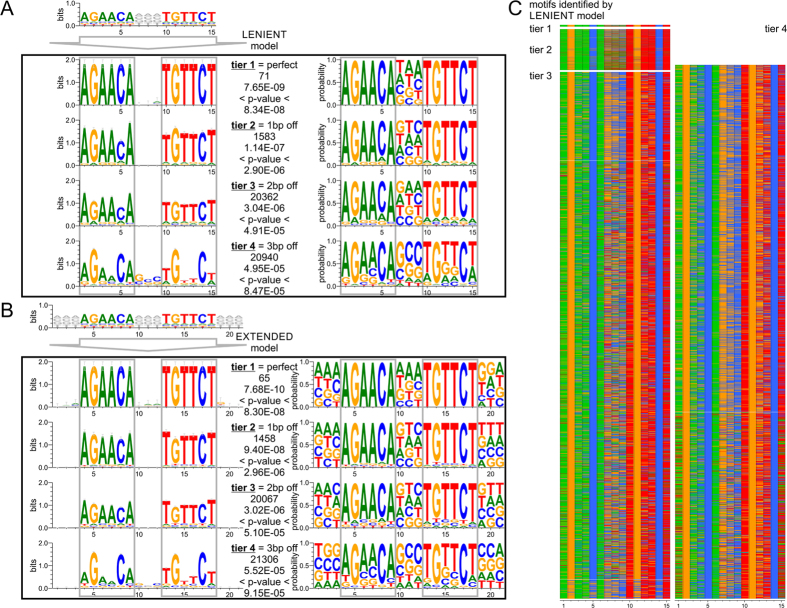
Identification and base-specific classification of AREs utilizing ChIP-Seq data. (**A**) ARE motifs discovered by ChIP-Seq data in combination with a lenient position site specific matrix model reflect the ideal 15mer motif of two hexamers with a 3mer spacer in its top hits. Additional tiers show up to three base pairs deviation off the ideal motif. (**B**) A search with an extended position site specific matrix model allows for insight into the environment of the core sequence motif. ARE logos on the left show the frequencies of the nucleotides scaled to the measure of conservation at each position while ARE logos on the right show the probability of a nucleotide being at that particular region. (**C**) Heatmap of discovered motifs by the lenient model shows conservation of G and C within each hexamer.

Nucleotide preference and deviation within a response element is critical in determining both the selectivity of transcription factors that bind to that sequence and the necessity for cooperating factors to assist with AR binding. The sequences detected by the ideal, lenient, half site, and extended PSSM model searches were quantified and sorted into tiers reflecting how much the sequence motifs deviated from an ideal ARE sequence (Supplementary Table S2). The ideal 15mer model of AGAACANNNTGTTCT had 71 AREs. The matches fell into tier 1, perfect motifs, with p-values below 8.34E-08. The lenient model identified the same matches in tier 1 and added less defined ARE into additional tiers. There were 71 AREs in tier 1 (perfect), 1583 matches in tier 2 (1bp off perfect), 20362 in tier 3 (2bp off perfect), and 20940 in tier 4 (3bp off perfect) with p-values below 4.94E-05 (Fig. 2A) (Supplementary Table S1 and Supplementary Table S2). The motifs showed conservation of G and C in positions two and five respectively in the ARE hexamers. In addition, there was increased GC content in the spacer region of the response element. The extended model largely agreed with the lenient model. In tier 4, there were 21306 motifs detected with p-values below 5.52E-05 (Fig. 2B). Regions neighboring the core hexamers as well as the spacer region had increased GC content. The conserved G and C of the hexamer in the least defined AREs of tier 4 were comparable to tier 4 of the lenient model. The overall decreased detection by the extended model in tiers 1–3 in comparison to the lenient model is attributed to the nucleotides of truncated motifs at the border of the ChIP-Seq peak as well as masked repeats in the genome which were blocked out of the search. Heatmaps of all detected motifs by the lenient model highlighted the observed GC nucleotide preferences in positions 2,5,7,8,9,11, and 14 (numbering of 15mer) in contrast to varying nucleotide content in the AT dominated positions (Fig. 2C). While tier 4 of the ARE full site requires agreement in at least 9 bases over a length of 15 bases including the 3mer spacer region, there are many examples reported where the entire half of the ARE full site is degenerate^55^. Lastly, to comprehensively describe the genome-wide coverage of AREs, tier 5, focuses on ARE half sites. Tier 5 ARE half sites show a perfect hexamer, while not complying with the requirements of tiers 1 through 4. By comparing genomic coordinates for ARE half sites and full sites, we made sure to create association with the lowest tier possible of any ARE in question. Model-based motif searches offer the possibility to further expand the degeneracy, for example by allowing imperfect ARE half sites. However, increasing numbers of degenerate motifs create limited genomic enrichment detecting almost every gene. The 5 tiers listed provided genome-wide coverage while recognizing functional relevant content. Model-based motif searches offer the possibility to further expand the degeneracy, for example by allowing imperfect ARE half sites. However, genome-wide searches with additional degeneracy did not generate other motifs than already detected by lower tiers. In total, we detected 42956 ARE full sites (tiers 1–4) and 79065 ARE half sites (tier 5).

### Genomic annotation and transcriptional regulation of ARE sites

**Figure 3:**
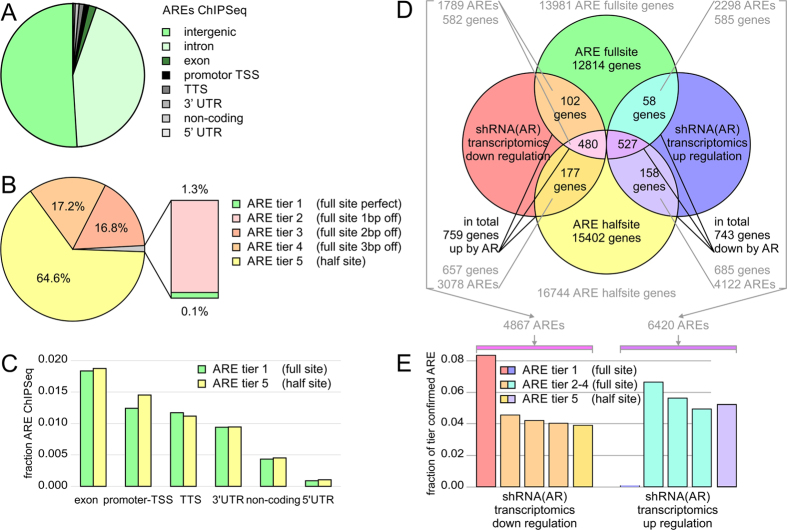
Genomic annotation and enrichment of AREs. (**A**) Identified AREs in ChIP-Seq experiment were annotated by genomic elements. (**B**) Classification of AREs into five tiers. There were 71 AREs in tier 1 (perfect palindromic ARE), 1583 matches in tier 2 (1 bp off perfect), 20593 in tier 3 (2 bp off perfect), 21031 in tier 4 (3 bp off perfect) and 79065 in tier 5 (half site) with p-values below 4.94E-05 in the search for motif matches. (**C**) Genomic location of ARE full and half sites. Intergenic and intronic locations are not shown. (**D**) Overlay of gene mapping of AREs identified by ChIP-Seq and transcriptomics experiments. Using this data we defined the group of 759 genes as positively regulated by AR activity (down in cell with shRNA knockdown of AR), and 743 genes as negatively regulated by AR activity. (**E**) AREs mapped by ChIP-Seq experiments were confirmed by transcriptomics experiment and matched to genes. AREs confirmed by transcriptomics experiment showed higher hit rate in better defined tiers of AREs. Hit rate plotted as fraction of tier confirmed by significant down or up regulation in stable shRNA knockdown experiment with p-values below 0.05. Different tiers of AREs suggest different effect on transcriptional outcome as well as necessity of modulation of weaker motifs by coordinating factors. Imperfect AREs make up majority of genome-wide recognition motifs.

Next, we sought to compare the functional content of different ARE tiers, in particular ARE half vs full sites. In agreement with the ChIP-Seq data, the majority of AREs falls into intergenic and intronic regions (Fig. 3A). While perfect AREs account only for a fraction of the AR recognition sites, tier 3 and 4 comprise 96% of the detected full site AREs (Fig. 3B). These higher tiered AREs approximate to about twice as many isolated half sites (tier 5) for every full site (tier 1–4). ARE-harboring regions within the ChIP-Seq data were annotated to the human genome, which allowed these regions to be categorized based on their relative location and distance to the nearest gene loci. Promoter or transcription start sites (TSS, by default defined as minus 1000 bp to plus 100 bp from the start of the precursor mRNA-coding gene locus) and transcription termination sites (TTS, by default defined as minus 100 bp to plus 1000 bp from the end of the mRNA) genomic annotation are defined as being within ± 5000 bp window of the ends of the gene-coding body. Intergenic regions were defined as the remaining regions outside the gene body of TSS and TTS. The intergenic regions (50.9%) accounted for the majority of the peaks in agreement with previous ChIP-Seq experiments using AR antibodies (Fig. 3A)^56^. 18693 (43.5%) of the AREs were annotated as intronic regions, 788 (1.8%) as exonic regions, 533 (1.2%) as TSS regions, 503 (1.2%) as TTS regions, 404 (0.9%) as 3′ UTR regions, 186 (0.4%) as non-coding regions, and 38 (0.1%) as 5′ UTR regions (Fig. 3C). ARE half sites show a significantly higher fraction of TSS annotation of (1.4%), in particular bi-directional promoters, than ARE full sites (1.2%) with a p-value below 10E-04, suggesting beneficial genomic proximity for weaker half sites. However, when annotated to gene bodies there appeared to be the same proportion of full and half site containing genes, despite the larger number of ARE half sites (Fig. 3D) (Supplementary Table S3). Each tier reflected this annotation and no significant overrepresentation of functional elements were detected in individual tiers, despite ARE half sites showed more locations associated with TSS than ARE full sites. The functional role of more than 120,000 AREs detected by our searches were tested against the transcriptional response of AR knockdown in a microarray experiment. The large number of AREs indicate that there are occurrences where multiple AREs are associated with a single gene. When compared to the transcriptomic data, in total 759 genes were transcriptionally down regulated and 743 genes were up regulated upon shRNA AR knockdown. When looking at the fraction of confirmed AREs per tier there is a higher hit rate in ARE tiers closer to the canonical sequence (Fig. 3E). Despite their lower abundance in the genome compared to ARE half sites, ARE full sites are able to generate a reliable transcriptional outcome in AR positive cells.

### Impact of somatic copy number alterations on genome-wide ARE utilization

**Figure 4:**
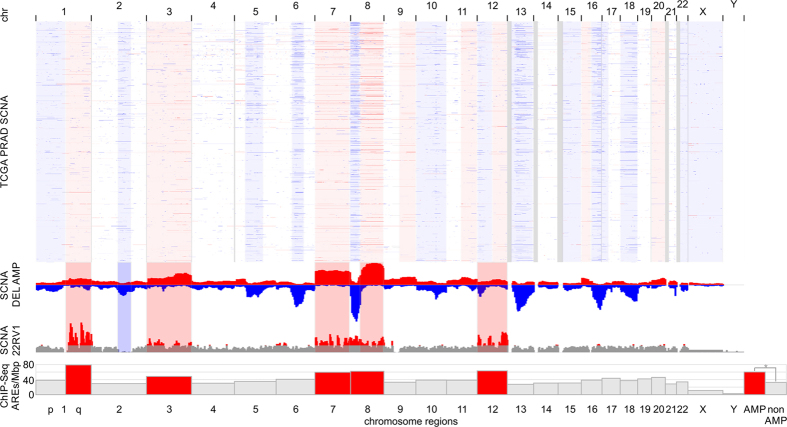
Copy number amplifications of the CWR22Rv1 cell line overlap with significant somatic copy number events in TCGA prostate adenocarcinoma patients and correlate with AR-ARE binding events by ChIP-Seq. Somatic copy number alteration (SCNA) profiles of 492 prostate adenocarcinoma (PRAD) patients in The Cancer Genome Atlas (TCGA) show broad arm-level events. Amplifications (AMP) are indicated in red; deletions (DEL) are indicated in blue. Low coverage next generation sequencing data of the CWR22Rv1 cell line reveals strong copy number amplification of chromosome arms 1q, 3p, 3q, 7p, 7q, 8p, 8q, 12p, and 12q. Utilization of androgen response elements by the androgen receptor is significantly elevated in amplified regions. Bar graph shows detected ARE-binding events by AR ChIP-Seq per megabase pair (Mbp).

Genotypic variation can modulate transcription factor binding, chromatin structure, and gene expression. In cancer progression, somatic copy number alterations (SCNAs) play critical roles by activating oncogenes and inactivating tumor suppressors^39^. In order to evaluate the significance of ARE utilization in the context of SCNAs, we determined recurring regions of copy number changes in 492 prostate adenocarcinoma (PRAD) patients in The Cancer Genome Atlas (TCGA) using the algorithm GISTIC. SCNA profiles of PRAD patients showed broad amplified arm-level events at 1q, 3p, 3q, 7p, 7q, 8p, 8q, 9q, 11q, 12q, 16p, 20p, and 20q and deleted regions at 1p, 6q, 8p, 10p, 10q, 12p, 13q, 15q, 16q, 17p, 18p, 18q, 22q, Xp, Xq, accompanied by focal events at 1p22, 1p31, 2q22, 3p13, 4q28, 5q11, 8p21, 11p11, 11q22, 12q24, 17q21, 19q13 (SCNA frequency more than 0.05; q-value less than 0.1) (Supplementary Table S4). Next, we determined SCNAs in the CWR22Rv1 cell line. Chromosome arms 1q, 3p, 3q, 7p, 7q, 8p, 8q, 12p, and 12q showed strong amplifications overlapping with SCNA regions identified in the TCGA PRAD cohort (Fig. 4). In order to assess if SCNAs modulate ARE binding, we determined the number of ChIP-Seq-detected AREs corrected for chromosome length. Regions with somatic copy number amplifications had significantly enhanced ARE utilization of 60.4 AREs/Mbp in contrast to euploidic genome regions of 32.3 AREs/Mbp with a p-value of 8.9e10-6 (Fig. 4). The amplified regions contained 5012 genes. In our ChIP-Seq and transcriptomic experiments 214 amplified genes classified as positive AR targets (down-regulation with shRNA knockdown) and showed exclusive enrichment of discrete pathways (p-value below 10e-03 and q-value below 0.1). Amplified and AR-responsive genes squalene epoxidase (SQLE, GeneBank: 6713), hydroxysteroid (17-beta) dehydrogenase 7 (HSD17B7, GeneBank: 51478), phosphomevalonate kinase (PMVK, GeneBank: 10654), lipoprotein lipase (LPL, GeneBank: 4023), v-myc avian myelocytomatosis viral oncogene homolog (MYC, GeneBank: 4609), NK3 homeobox 1 (NKX3-1, GeneBank: 4824), ELK4, ETS-domain protein (SRF accessory protein 1) (ELK4, GeneBank: 2005), PTK2B protein tyrosine kinase 2 beta (PTK2B, GeneBank: 2185), and zinc finger and BTB domain containing 10 (ZBTB10, GeneBank: 65986) are pathway members of the androgen response, steroid biosynthesis, and cholesterol homeostasis. In addition, amplified and AR-regulated genes showed enrichment in MTORC1 signaling, DNA replication, cell cycle, MYC targets, mismatch repair, homologous recombination, nucleotide excision repair, epigenetic regulators, and pathways in cancer. The detected ARE recognition by the androgen receptor displays a potential mechanism how SCNAs get translated to a functional, oncogenic level in prostate cancer.

### Comparison of ARE utilization in androgen-insensitive and androgen-responsive prostate cancer cell lines

**Figure 5:**
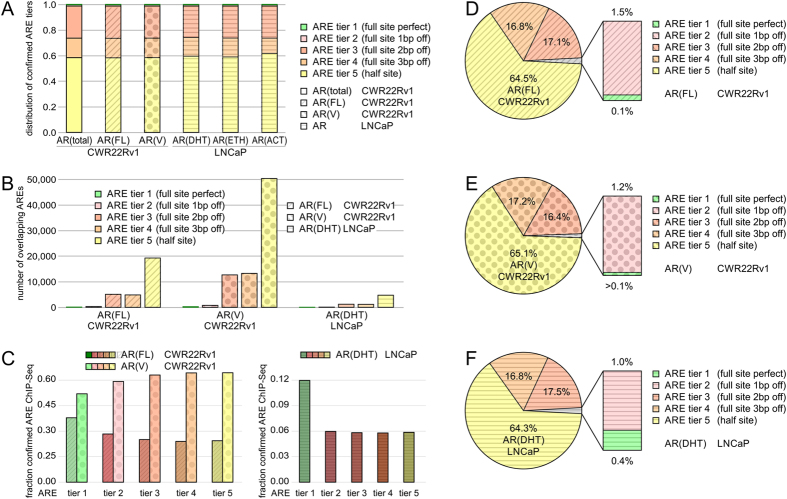
Utilization of androgen response elements in androgen-dependent LNCaP and androgen-independent CWR22Rv1 cellular models of prostate cancer. ARE binding by ChIP-Seq was assessed in the CWR22Rv1 cell line (total binding; binding by full-length androgen receptor, AR(FL); binding by variant androgen receptor, AR(V)) and in the LNCaP cell line (5α-dihydrotestosterone treatment, AR(DHT); ethanol treatment, AR(EtOH); functionally active steroid-bound AR corrected for EtOH background, AR(ACT)). (**A**) Comparison of AREs detected and confirmed by ChIP-Seq in AR(FL), AR(V), and AR(DHT) specimen. (**B**) Distribution of confirmed AREs by ChIP-Seq in CWR22Rv1 and LNCaP cells. (**C**) Fraction of confirmed AREs by ChIP-Seq in CWR22Rv1 and LNCaP cells. Pie charts visualizing distribution of ARE tiers detected and confirmed by ChIP-Seq in D) AR(FL) in CWR22Rv1 cells, (**E**) AR(V) in CWR22Rv1 cells, and (**F**) AR(DHT) in LNCaP cells. Tier 1 is highlighted in green, tier 2 in magenta, tier 3 in red, tier 4 in orange, tier 5 in yellow. ChIP-Seq analysis for AR(FL) is shaded with tilted lines, AR(V) with dots, and AR(DHT) with horizontal lines.

Androgen-insensitive cell lines, such as CWR22Rv1, express AR(FL) and AR splice variants^28^. In contrast, androgen-responsive prostate cancer models expressing exclusively AR(FL), such as LNCaP, offer insights into steroid-dependent gene regulation^30^. We applied the established model-based ARE annotation to condition-specific CWR22Rv1 and LNCaP ChIP-Seq samples and assessed ARE utilization dependent on AR splice isoforms as well as 5α-dihydrotestosterone treatment (Fig. 5). ARE binding by ChIP-Seq was assessed in the CWR22Rv1 cell line for total AR binding, AR(total), for binding to full-length androgen receptor, AR(FL), for binding by variant androgen receptor, AR(V), in the LNCaP cell line for 5α-dihydrotestosterone treatment, AR(DHT), for ethanol treatment, AR(EtOH), and for functionally active steroid-bound AR corrected for EtOH background AR(ACT). All quantified conditions showed a similar distribution of ARE tiers 1–5 of about 0.001, 0.017, 0.244, 0.150, and 0.588, respectively (Fig. 5A). The data validates that utilization of perfect ARE tier 1 is a rare event, independent of AR splice-variants or steroid condition. Cross-validation of ChIP-Seq experiments of AR(total) vs AR(FL) confirmed 30,022 AREs assigned to AR(FL)-binding in the CWR22Rv1 cell line (Fig. 5B). AR(V) isoforms with 78,350 ARE ChIP-Seq events bind to DNA autonomous of full-length androgen receptor in the absence of androgen and modulate a unique set of genes that is not regulated by full-length androgen receptor (Fig. 5B). In contrast, AR(DHT) in the LNCaP cell line displayed a set of 7,361 AREs common to the CWR22Rv1 cell line (Fig. 5B). Next, we quantified the fraction of ARE tiers confirmed in overlapping ChIP-Seq experiments. AR(FL) showed an incremental reduced fraction with higher, less specific ARE tiers in the CWR22Rv1 cell line, while AR(V) isoforms had a stronger overlap with more degenerate motifs (Fig. 5C). The AR(DHT) LNCaP condition showed a trend similar to AR(FL) in the CWR22Rv1 cell line (Fig. 5C). The distribution of ARE tiers with AR splice-variants or with steroid treatment in the two tested PRAD cell lines showed most variation in the perfect AREs of tier 1. Notably, the frequency of perfect AREs correlates with AR specificity and increases from AR(V) isoforms to AR(FL) in the CWR22Rv1 cell line and quadruples in the AR(DHT) LNCaP condition (Fig. 5D–F). All evaluated conditions showed high agreement utilization of ARE tiers 3–5, half sites and imperfect full sites (Fig. 5D–F).

### Network of transcriptional cooperation of the androgen receptor

**Figure 6:**
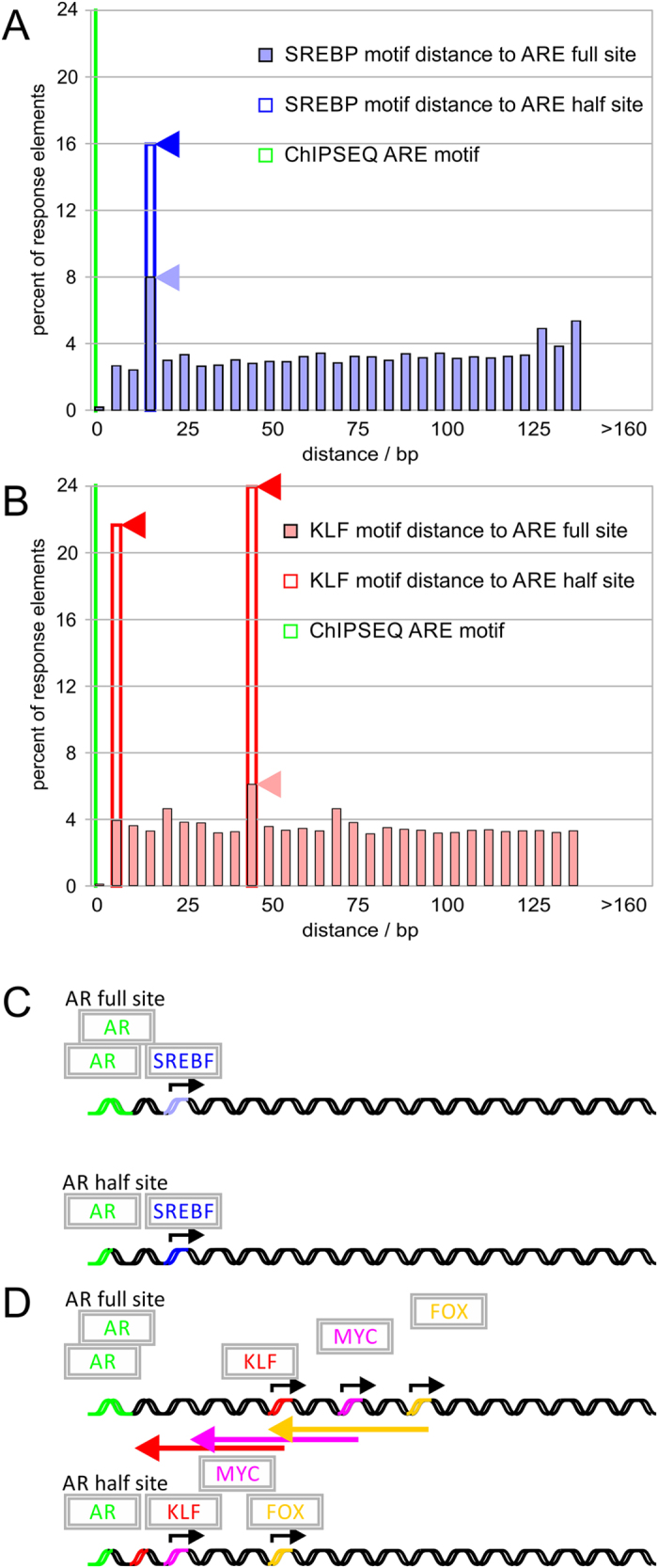
Enhancement of androgen response elements by cooperation of the androgen receptor with other transcription factors. Transcription factor complexes were inferred from ChIP-Seq data using spaced motif analysis in reference to detected AR binding sites (green). The affinity of weaker ARE transcription factor sites can be enhanced by cooperation with other transcription factors. (**A**) SREBF transcription factor family (blue) shows an increase in the percent of response elements found 15 bp from the ARE half site. (**B**) KLF transcription factor motif family (red) shows an increased fraction bound in AR ChIP-Seq signals. In addition, peaks of KLF motifs in 5 bp and 45 bp distance to ARE half sites shows increased proximity compared to association with ARE full sites. The weaker ARE half-site shows strong cooperation with (**C**) SREBF and closer motif distance with (**D**) KLF, MYC, and FOX, transcription factor families with the AR.

We next sought to identify potential transcription factors that would cooperate with the AR to regulate gene expression. Using the Jaspar motif database, we grouped significant transcription factor logos within a window of ±160 bp from the ARE with a p-value of less than 0.05. Top hits included forkhead box (FOX), Krüppel-like factors (KLF), basic helix-loop-helix (BHLH), sterol regulatory element binding factor (SREBF), and v-myc avian myelocytomatosis viral oncogene homolog (MYC) families of transcription factors. Interestingly, several members of cooperating transcription factor families showed amplifications at the copy number level in the CWR22RV1 cell line as well as in TCGA PRAD patients. Detected somatic amplifications of transcription factors were maintained at the transcriptional level. Somatic copy number amplifications on chromosome 3 included Krüppel-like family member Krüppel-like factor 15 (KLF15, GeneBank: 28999). Similarly, amplified regions on chromosome 7 included transcription factors v-myc avian myelocytomatosis viral oncogene homolog (MYC, GeneBank:4609) and forkhead box K1 (FOXK1, GeneBank: 28999). We then organized recorded distances of detected, enriched, and/or amplified transcription factor families to the ARE into histograms with 5bp bins and analyzed spacing of the transcription factor motifs. We noticed an increase of detected transcription factor motifs at 15 bp for KLF, and 45 bp for SREBF-related transcription factors between full and half site ARE (Fig. 6A,B). This suggests that for the AR to recognize any weaker half site response element, cooperation of other transcription factors might be required (Fig. 6C,D). Distance of KLF, MYC, or FOX motifs to ARE half sites was reduced in comparison with distance to ARE full sites (Fig. 6D). Taken together, the transcription factor network analysis (top hits in motif enrichment with p-values below 0.05) suggests that KLF, MYC, FOX, and SREBF families of transcription factors have the ability to utilize motifs in the cistrome of AREs and to cooperate with the AR.

### Quantifying the transcriptional response between full and half site ARE with KLF

**Figure 7:**
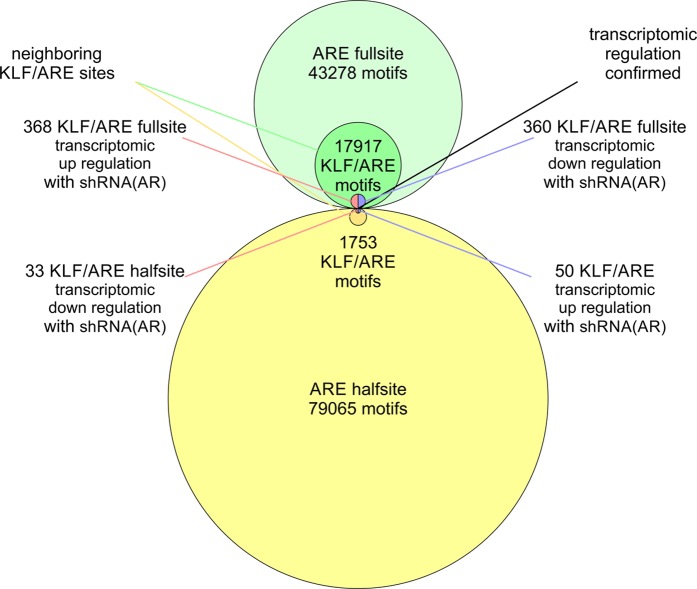
Synergy of ARE and KLF motifs in androgen receptor-mediated transcriptional responses. Quantification of KLF sites with respect to detected ARE full sites and half sites. 17917 full sites coincide with KLF sites. 25 fell into tier 1, 611 into tier 2, 8067 into tier 3, and 9214 into tier 4. Stronger cooperation of ARE full sites with KLF motifs result in larger transcriptional response (368 and 360 genes up and down in cells with shRNA knockdown of AR, respectively) in contrast to ARE half sites (33 and 50 genes up and down, respectively).

Next, we characterized transcription factor cooperation of the AR with the Krüppel-like family. We quantified the number of KLF sites with respect to the detected ARE tiers to evaluate effect of transcriptional cooperation with respect to motif degeneracy (Supplementary Table S5 and Supplementary Table S6). We found that 17917 full sites coincide with KLF sites. 25 fell into tier 1, 611 into tier 2, 8067 into tier 3, and 9214 into tier 4. When comparing between detected full and half sites there was a stronger cooperation of the ARE full sites with KLF motifs resulting in larger transcriptional response with 360 and 368 genes up and down regulated in contrast to ARE half sites, which had 50 and 33 genes up and down regulated (Fig. 7). Despite a larger number of weaker ARE half sites found in the proximity of KLF motifs, stronger AREs next to KLF motifs resulted in a larger transcriptional response. Genes associated with pathways in cancer as well as TP53 signaling were enriched in genes with KLF and ARE full site motifs with p-values below 0.05 (Supplementary Table S5). For KLF and ARE half site motifs pathways of extracellular matrix-receptor interaction and focal adhesion with p-values below 0.001 were found. Consequently, the data suggests that KLF may modulate the binding of AR with both weaker and stronger AREs, which control genes with distinct function.

### Functional enrichment of androgen receptor binding sites

Gene set enrichment analysis of identified putative AR target genes revealed several functional clusters. In addition to the identified 759 and 743 genes confirmed by ChIP-Seq binding as well as transcriptional activity, we tested all ARE full site and half site tiers for pathway enrichment (Supplementary Table S5). Gene sets corresponding to full site AREs with activating gene expression (582 genes as positively regulated by AR activity with ARE full or half sites; 102 with exclusively full sites) revealed 41 including 21 exclusive pathways significantly enriched with p-values below 0.05. For half site AREs (585 genes as positively regulated by AR activity with ARE full or half sites; 53 with exclusively full sites) 39 including 12 exclusive pathways were found with p-values below 0.05. Pathways in both sets included DNA replication, cell cycle control, and metabolic pathways. Of particular interest were pathways that were exclusively assigned to ARE full sites or half sites. The set of AR target genes with ARE full sites focused on pyrimidine metabolism, terpenoid backbone biosynthesis, one-carbon metabolism, glycine, serine and threonine metabolism with p-values below 0.001. The metabolic program—framed by genes with full site AREs—supports proliferative functions required for cellular maintenance. In contrast, the set of AR target genes with ARE half sites included steroid biosynthesis, terpenoid backbone biosynthesis, peroxisome, pentose phosphate pathway, glycerolipid metabolism, and mitogen activated protein kinase signaling pathway with p-values below 0.001. An enrichment of genes containing ARE half sites involved in lipid and steroid biosynthesis could point to the gender-, development-, and tissue-specific control in prostatic differentiation. Therefore, the gene set enrichment analysis suggests that AR targets genes controlled by ARE full sites and/or ARE half sites have common proliferative functions but also distinct biological functions involved in lipid metabolism.

## Discussion

We refined AR-binding and AREs in AR-positive but androgen-insensitive CWR22Rv1 prostate cancer cells using ChIP-Seq and motif-guided genome-wide analysis. The independence of androgen ligand for AR activity makes the CWR22Rv1 cell line a favorable model for genome-scale characterization of AR binding events in prostate cancer. Therefore, one can accomplish a comprehensive, unprecedented picture of the ARE by using ChIP-Seq analysis of AR-specific immunoprecipitation. We classified AREs according to their degeneracy and their transcriptional involvement. We quantified ARE utilization in response to somatic copy number amplifications, AR splice-variants, and steroid treatment. Our AR ChIP-Seq mapping shows that a majority of AREs are imperfect AREs with several base pairs deviating from the canonical palindromic 15 mer ARE full site. Our results fit in with previous assessments that AR DNA binding is possible despite base pair deviation from the canonical ARE full site sequence^23^. Although imperfect AREs make up 99.9% of the motifs, the degree of degeneracy correlates negatively with validated transcriptional outcome. Weaker AREs, particularly ARE half sites, benefit from neighboring motifs or cooperating transcription factors in regulating gene expression. In addition, ARE half sites showed enrichment of mitogen activated protein kinase signaling required for stimulation of proliferation. Therefore, the ability to regulate weaker AREs carries weight for prostatic development and oncogenic control^23^.

Somatic copy number alterations of TCGA prostate cancer patients correlated with amplifications of chromosome arms 1q, 3p, 3q, 7p, 7q, 8p, 8q, 12p, and 12q observed in the cellular CWR22Rv1 prostate cancer model. Aneuploidy of the CWR22Rv1 model was recognized early on during the genetic characterization of the metastatic cell line^27^. Most SCNA studies in prostate cancer have focused on AR gene amplification found in the majority (87%) of CRPC tumors^57^,^58^,^59^. The number of recognized ARE sites increases with the level of AR expression^60^. A study in LNCaP cells described AR recruitment to amplified chromosomal regions found in metastatic PRAD implicating AR co-amplification with SCNAs^61^. Similarly, our SCNA data showed a higher density of ARE binding events in amplified regions suggesting that ARE recognition and amplification might play a role in carrying SCNAs to a functional level in prostate cancer.

We applied genome-wide model-based motif searches to different prostate cancer models and investigated ARE occurrence dependent on AR-splice variants and steroid regulation. Derived from an androgen-dependent CWR22 mouse xenograft that relapsed during androgen deprivation, the CWR22Rv1 prostate cancer cell line is androgen-insensitive, expresses different AR isoforms^4^,^8^,^9^,^27^,^62^, and is expected to display enhanced ARE recognition. ChIP-Seq data on the CWR22Rv1 cell line revealed 122021 AREs (32.0% ARE full sites; 68.0% ARE half sites). 88.5% of the detected AREs overlapped with ChIP-Seq data of published experiments after processing using the same bioinformatics workflow^28^. AR(V) isoforms recognize 2.6 times more AREs than AR(FL) in the isoform-specific ARE characterization of the CWR22Rv1 cell line. In contrast, the LNCaP cell line serves as a model for primary prostate tumors that are responsive to ADT therapy^29^. Using genome-wide model-based motif searches, the LNCaP cell line under testosterone-treatment displayed 6.0% overlap with the CWR22Rv1 cell line resembling the fraction of AREs recognized under androgen stimulation^30^. The fraction of AREs activated under androgen stimulation in LNCaP is small compared to the total number of AREs recognized in the CWR22Rv1 model. Dihydrotestosterone-activated AR response resembles a narrow, well-defined, physiological gene-expression program required for prostatic function^30^,^63^. In contrast, the altered and enhanced spectrum of AREs assigned to shortened, non-specific CWR22Rv1 AR(V) isoforms mediates an oncogenic gene expression program that is able to circumvent androgen deprivation, support continued proliferation, and drive CRPC^8^,^20^,^28^.

AREs can be modulated by cooperation with other transcription factors, which can compensate for missing canonical contacts of imperfect AREs and nevertheless result in successful gene expression events^23^,^55^. The cistrome of investigated AREs actively participates in the AR controlled gene expression within CRPC^64^,^65^. A model of susceptibility to cooperation of weak AREs with neighboring transcription factors has already been confirmed for different transcriptional networks with the AR^55^,^23^. In our motif searches, the validated AR-cooperating transcription factor FOXA1 displayed motif enrichment, differential up-regulation, and high tumor expression, serving as test and validation data point. Overexpression of FOXA1 promotes cell cycle progression, CRPC signature, and has been described as playing a crucial role in assisting AR site recognition for weaker binding sites by creating excessive open chromatin sites^66^,^67^,^68^. The transcriptional program required for prostate-specific gene expression of steroid biosynthesis enzymes is distinctly enriched in ARE half sites and consistent with modular and tightly regulated tissue-specific control^69^,^70^. In PRAD, cooperation of SREBF and AR signaling has been linked to progression of prostate cancer cells^71^. SREBF targeted gene expression drops drastically after androgen deprivation therapy, but their transcription program re-emerges upon reactivation of the AR transcriptional program manifesting the hypothesis of transcriptional cooperation^72^. In addition, SREBF has been shown to be recruited with the AR to target gene promoters^73^. In the Krüppel-like transcription factor family, motif enrichment and somatic amplification of chromosome 3 suggest KLF15 as candidate transcription factor to support the AR. While the genomic data provide strong evidence for cooperation, experimental validation will be required to solidify this finding. Other cellular models have linked KLF15 overexpression with enhanced recruitment of nuclear receptors^74^,^75^. Previous analyses of the KLF15 promoter detected AREs suggesting hormonal control of its transcription^76^. Development- and cell-specific expression of KLF isoforms may modulate AR signaling, where AREs and KLF motifs fall in close proximity. Taken together, promiscuous recognition of androgen receptor cognate sites guided by transcriptional cooperation may allow for dynamic, tissue-specific regulation.

A predominant signature of imperfect AREs is the GC content of the 3 bp spacer as well as the flanking regions of extended motifs. The GC content is richest in higher, less perfect tiers of AREs. CpG dinucleotides within the genome enable the cell to control ARE availability by DNA 5′-cytosine methylation^77^,^78^. Actively transcribed genomic regions, in particular promoter or transcription factor cognate sites, tend to have less DNA methylation^79^,^80^,^81^. Therefore, weaker AREs are subject to control of gene expression by the dynamic equilibrium of histone and DNA methyltransferases and demethylases^82^. Conformational change of the protein structure plays an important role in the ambiguity of ARE recognition. The AR is able to recognize weaker sites by first binding to a high affinity AGAACA site followed by a strong conformation change in the protein and possibly in the ARE^83^,^84^. While analyzing the different tiers of the ARE in our data we found that the 1–3 nucleotides would be off in only one of the hexamers recognized by the homodimer DBD. Additionally, as we progressed to the higher tiers the G and C in positions 2 and 5 in the AR hexamer remained conserved. Our work suggests that ARE full sites are able to bind to and recognize weaker sites through binding of a stronger half site within the full site and that the weaker site is more dependent upon the G and C residues for complete homodimer binding. Further, the GC content is also important for the spectrum of cooperating transcription factors with the AR. The KLF family recognizing GC/GT boxes has been implicated in regulation of oncogenic expression signatures in LNCaP and PC3 prostate cancer cell lines^85^,^86^. Transcription factors cooperating with the AR form an important regulatory hierarchy governing androgen-dependent gene expression in normal as well as malignant prostate tissue and offer potential new opportunities for therapeutic intervention.

## Conclusion

Our data refined the recognition of ARE sequences within CWR22Rv1 and LNCaP prostate cancer cell lines. We expanded the nucleotide specificity of the ARE, identified potential modes of regulation these response elements are subject to, and outlined a protocol for identifying coordinating transcription factors to assist with weaker site recognition. While a major disadvantage remains in possible false-negatives being identified in computationally predicted sites, future experimental verification will have to determine if these response elements play a role in gene regulation. Future ChIP-Seq studies could look into prominent histone modifications accompanying detected response element sites within prostate cancer and facilitate insight how chromatin alteration affects AR gene targeting. Importantly, we identified significant differences in the genomic landscape of ARE full and half sites. Despite the fact that ARE half sites outnumber ARE full sites by 2-fold, stronger ARE were more frequently confirmed at the transcriptional level than weaker AREs. Nevertheless, weaker AREs are affected by AR expression or regulation, and may have strong functional impact by multiplicity and/or genomic proximity. ARE impact depends on somatic alterations, motif-receptor-binding specificity, tissue-specific lineage, cooperating factors (e.g. FOX, SREBF, MYC, KLF), distance to neighboring motifs, AR-splice variants, and steroid regulation.

## Additional Information

**How to cite this article**: Wilson, S. *et al*. Refinement of the androgen response element based on ChIP-Seq in androgen-insensitive and androgen-responsive prostate cancer cell lines. *Sci. Rep.* **6**, 32611; doi: 10.1038/srep32611 (2016).

## Supplementary Material

Supplementary Tables 1-7

Supplementary Information
