# Supplementary material for: Refinement of the androgen response element based on ChIP-Seq in androgen-insensitive and androgen-responsive prostate cancer cell lines
Source: Sci Rep. 2016 Sep 14;6:32611. doi: 10.1038/srep32611 (PMC5021938; doi:10.1038/srep32611)

Refinement of the androgen response element based on ChIP-Seq
in androgen-insensitive and androgen-responsive prostate cancer cell lines

Systems Biology and Cancer Metabolism,
Program for Quantitative Systems Biology,
University of California Merced, Merced, CA 95343, USA
Stephen Wilson, Jianfei Qi#, Fabian V. Filipp**
Address: 2500 North Lake Road, Merced, CA 95343, USA
Phone: +1-858-349-0349
e-mail: filipp@ucmerced.edu
systemsbiology.ucmerced.edu

SREP-16-06847, 2016
http://dx.doi.org/10.1038/srep32611
http://www.nature.com/articles/srep

SI	Supplementary Tables	Tab
	Glossary	Glossary
SI_01	Supplementary Table 1	SI01_ARE_LOGO_PSSM
SI_02	Supplementary Table 2	SI02_CHIPSEQ_ARE_Annotation
SI_03	Supplementary Table 3	SI03_CHIPSEQ_Genelist
SI_04	Supplementary Table 4	SI04_SCNA
SI_05	Supplementary Table 4	SI04_CHIPSEQ_PATHWAYS
SI_05	Supplementary Table 5	SI05_CHIPSEQ_ARE_KLF_MOTIFS

	Content
	Explanation of biological nomenclature and abbreviations
	Position site specific matrix (PSSM) models describe a search pattern for motif discovery.
	ARE locations identified by PSSM-based motif models.
	Genomic annotation and gene mapping of AREs.
	Somatic copy number alteration (SCNA) profiles in prostate cancer.
	Gene set enrichment analysis of identified putative AR target genes.
	Genomic annotation and overlap of KLF motifs and AREs.

SI	Supplementary Legends	Tab
SI_01	Supplementary Table 1	SI01_ARE_PSSM
	Position site specific matrix (PSSM) models describe a search pattern for motif discovery.	
	Nucleotide frequency matrices used as a criteria for the FIMO MEME suite version 4.04 for searching sequenced data for motif sequences. Currently Jaspar matrix what is what is used to predict AREs found in sequenced data. However this matrix poorly predicts canonical full site ARE models and subsequent ARE models were needed to predict ARE patterns. The ideal model was designed for finding canonical ARES, while the lenient model allowed for more degenerate canonical AREs to be found, and the extended model allowed for nucleotides neighboring the AREs outside genomic masked regions to be identified. The refined, experimentally derieved ARE full and half sites are provided in transposed logo format and deposited into the transcription factor databases.	
SI_02	Supplementary Table 2	SI02_CHIPSEQ_ARE_Annotation
	ARE locations identified by PSSM-based motif models.	
	Results by the FIMO MEME suite v. 4.04 tool are listed in a table format by the motif model scanned, genomic coordinates, p value, and motif.	
SI_03	Supplementary Table 3	SI03_CHIPSEQ_Genelist
	Genomic annotation and gene mapping of AREs.	
	Discovered ARE sequences were tagged with their genomic coordinates, the wig-bed region ARE is found, the genomic annotation, gene symbol, distance to nearest TSS, and microarray list.	
SI_04	Supplementary Table 4	SI04_SCNA
	Somatic copy number alteration (SCNA) profiles in prostate cancer.	
	SCNA profiles of the CWR22Rv1 prostate cancer line and 492 prostate adenocarcinoma (PRAD) patients in The Cancer Genome Atlas (TCGA).	
SI_05	Supplementary Table 5	SI05_CHIPSEQ_PATHWAYS
	Gene set enrichment analysis of identified putative AR target genes.	
	KEGG pathways with an overrepresentation of putative AR target genes. Significance of KEGG pathway is dependent upon the input size, and the number of genes found within the specific KEGG pathway.	
SI_06	Supplementary Table 6	SI06_CHIPSEQ_ARE_KLF_MOTIFS
	Genomic annotation and overlap of KLF motifs and AREs.	
	Discovered KLF motifs were matched with genomic coordinates of AREs confirmed by ChIPSeq as well as transcriptomic experiments.	

Abbreviation	Expression
ADT	androgen deprivation therapy
AR	androgen receptor, GeneBank: 367
ARE	androgen-response element
AR-FL	AR full-length
AR-V	AR splice variant
BHLH	basic helix-loop-helix
cDNA	Complementary DNA
ChIP	chromatin immunoprecipitation
ChIPSeq	ChIP in combination with next generation sequencing
CRPC	castration-resistant prostate cancer
CWR22Rv1	human prostate carcinoma epithelial cell line derived from CWR22 xenograft
DBD	DNA binding domain
DREME	discriminative regular expression motif elicitation
FIMO	find individual motif occurrences
FOX	forkhead box
KLF	Krüppel-like factors
LBD	ligand-binding domain
LIMMA	linear models for microarray analysis
MACS	model-based analysis of ChIPSeq
MEME	maximization for motif elicitation
NaCl	sodium chloride
NTD	N-terminal transactivation domain
PPIA	peptidylprolyl isomerase A, cyclophilin A, GeneBank: 5478
PRAD	prostate adenocarcinoma
PSSM	position site specific matrix
qRT-PCR	quantitative real time polymerase chain reaction
RIPA	radio-immunoprecipitation assay
RNaseA	ribonuclease A
SDS	sodium dodecyl sulfate
SDS-PAGE	sodium dodecyl sulfate-polyacrylamide gel electrophoresis
SPAMO	spaced motif analysis
SREBF	sterol regulatory element binding factor
TCGA	The Cancer Genome Atlas
Tris-HCl	trisaminomethane hydrochloride
TSS	transcription start sites
TTS	transcription termination sites
VSN	variance stabilization

ARE full site Experimental transcription factor logo deposited under accession AR in Jaspar, accession ANDR_HUMAN in HOCOMOCO, accession M08907, TRANSFAC, 2016.3 release.

Position	A	C	G	T
1	33065	730	3963	5198
2	1908	1316	38778	954
3	31476	4778	5110	1592
4	28126	7971	4545	2314
5	2064	37906	1955	1031
6	35865	3351	933	2807
7	7612	9703	17624	8017
8	8768	12839	12618	8731
9	8115	17543	9723	7575
10	2955	931	3413	35657
11	986	1978	37831	2161
12	2319	4317	8163	28157
13	1559	4888	4945	31564
14	863	38918	1269	1906
15	5263	3993	698	33002

ARE half site Experimental transcription factor logo deposited under accession M08908 in TRANSFAC, 2016.3 release.

Position	A	C	G	T
1	79065	0	0	0
2	0	0	79065	0
3	79065	0	0	0
4	79065	0	0	0
5	0	79065	0	0
6	79065	0	0	0
7	10444	20556	37506	10559
8	17086	23381	26668	11930
9	16723	21819	28641	11882
10	14815	27577	26222	10451
11	23595	19882	16954	18634
12	21666	17471	20697	19231
13	22489	17155	21526	17895
14	21919	15893	21401	19852
15	23017	17963	19774	18311

Meme PSSM

Position	A	C	G	T
1	0.778	0.000	0.111	0.111
2	0.074	0.000	0.889	0.037
3	0.630	0.074	0.296	0.000
4	0.074	0.741	0.000	0.185
5	0.074	0.593	0.074	0.259
6	0.630	0.074	0.296	0.000
7	0.037	0.444	0.444	0.074
8	0.370	0.333	0.000	0.296
9	0.111	0.370	0.407	0.111
10	0.222	0.000	0.111	0.667
11	0.037	0.037	0.926	0.000
12	0.444	0.037	0.407	0.111
13	0.185	0.037	0.778	0.000
14	0.037	0.815	0.111	0.037
15	0.481	0.185	0.000	0.333

Dreme PSSM

Position	A	C	G	T
1	0.000	0.000	0.000	1.000
2	0.000	0.000	1.000	0.000
3	1.000	0.000	0.000	0.000
4	0.000	0.000	1.000	0.000
5	1.000	0.000	0.000	0.000
6	1.000	0.000	0.000	0.000
7	0.000	0.740	0.000	0.340
8	1.000	0.000	0.000	0.000

IDEAL MODEL

Position	A	C	G	T
1	1.000	0.000	0.000	0.000
2	0.000	0.000	1.000	0.000
3	1.000	0.000	0.000	0.000
4	1.000	0.000	0.000	0.000
5	0.000	1.000	0.000	0.000
6	1.000	0.000	0.000	0.000
7	0.250	0.250	0.250	0.250
8	0.250	0.250	0.250	0.250
9	0.250	0.250	0.250	0.250
10	0.000	0.000	0.000	1.000
11	0.000	0.000	1.000	0.000
12	0.000	0.000	0.000	1.000
13	0.000	0.000	0.000	1.000
14	0.000	1.000	0.000	0.000
15	0.000	0.000	0.000	1.000

LENIENT MODEL

Position	A	C	G	T
1	0.700	0.100	0.100	0.100
2	0.100	0.100	0.700	0.100
3	0.700	0.100	0.100	0.100
4	0.700	0.100	0.100	0.100
5	0.100	0.700	0.100	0.100
6	0.700	0.100	0.100	0.100
7	0.250	0.250	0.250	0.250
8	0.250	0.250	0.250	0.250
9	0.250	0.250	0.250	0.250
10	0.100	0.100	0.100	0.700
11	0.100	0.100	0.700	0.100
12	0.100	0.100	0.100	0.700
13	0.100	0.100	0.100	0.700
14	0.100	0.700	0.100	0.100
15	0.100	0.100	0.100	0.700

EXTENDED MODEL

Position	A	C	G	T
1	0.250	0.250	0.250	0.250
2	0.250	0.250	0.250	0.250
3	0.250	0.250	0.250	0.250
4	0.700	0.100	0.100	0.100
5	0.100	0.100	0.700	0.100
6	0.700	0.100	0.100	0.100
7	0.700	0.100	0.100	0.100
8	0.100	0.700	0.100	0.100
9	0.700	0.100	0.100	0.100
10	0.250	0.250	0.250	0.250
11	0.250	0.250	0.250	0.250
12	0.250	0.250	0.250	0.250
13	0.100	0.100	0.100	0.700
14	0.100	0.100	0.700	0.100
15	0.100	0.100	0.100	0.700
16	0.100	0.100	0.100	0.700
17	0.100	0.700	0.100	0.100
18	0.100	0.100	0.100	0.700
19	0.250	0.250	0.250	0.250
20	0.250	0.250	0.250	0.250
21	0.250	0.250	0.250	0.250

Half site PSSM

Position	A	C	G	T
1	0.250	0.250	0.250	0.250
2	0.250	0.250	0.250	0.250
3	0.250	0.250	0.250	0.250
4	0.250	0.250	0.250	0.250
5	0.700	0.100	0.100	0.100
6	0.100	0.100	0.700	0.100
7	0.700	0.100	0.100	0.100
8	0.700	0.100	0.100	0.100
9	0.100	0.700	0.100	0.100
10	0.700	0.100	0.100	0.100
11	0.250	0.250	0.250	0.250
12	0.250	0.250	0.250	0.250
13	0.250	0.250	0.250	0.250
14	0.250	0.250	0.250	0.250

As reference position-shifted PSSM based on outdated database entry MA0007.2

Position	A	C	G	T
1	0.560	0.100	0.270	0.070
2	0.580	0.000	0.420	0.000
3	0.000	0.000	1.000	0.000
4	0.580	0.120	0.110	0.200
5	1.000	0.000	0.000	0.000
6	0.000	0.990	0.010	0.000
7	0.930	0.000	0.010	0.060
8	0.130	0.270	0.360	0.240
9	0.390	0.190	0.190	0.230
10	0.300	0.290	0.290	0.120
11	0.290	0.010	0.030	0.670
12	0.000	0.050	0.950	0.000
13	0.200	0.110	0.060	0.630
14	0.240	0.220	0.220	0.330
15	0.140	0.630	0.030	0.200

ARE ch	ARE Start	ARE Stop	Fimo P-valu	ARE Seque	Tier	Genomic Lo	Gene Symb	Distance to TSS	Reg	
chr6	35569858	35569872	7.65E-09	AGAACAGC	Tier one	intron(NM_	MIR5690	62701	0	0
chr7	1.27E+08	1.27E+08	7.65E-09	AGAACAGC	Tier one	intron(NR_	MIR592	-22995	0	0
chr1	83223955	83223969	3.56E-08	AGAACACC	Tier one	Intergenic	LPHN2	957880	0	0
chr1	84741353	84741367	3.56E-08	AGAACAGA	Tier one	Intergenic	SAMD13	-22689	0	0
chr10	35222735	35222749	3.56E-08	AGAACATG	Tier one	Intergenic	PARD3-AS1	118046	0	0
chr10	97123229	97123243	3.56E-08	AGAACAGC	Tier one	intron(NM_	PDLIM1	-72331	0	0
chr11	32134973	32134987	3.56E-08	AGAACAGC	Tier one	Intergenic	RCN1	22503	0	0
chr12	1919290	1919304	3.56E-08	AGAACATG	Tier one	intron(NM_	LRTM2	-10136	0	0
chr12	92424612	92424626	3.56E-08	AGAACATC	Tier one	intron(NR_	C12orf79	110870	0	0
chr13	31561664	31561678	3.56E-08	AGAACACA	Tier one	Intergenic	TEX26	54837	0	0
chr16	24979474	24979488	3.56E-08	AGAACAGC	Tier one	intron(NM_	ARHGAP17	47218	0	0
chr16	69286348	69286362	3.56E-08	AGAACACC	Tier one	intron(NM_	VPS4A	-58932	0	0
chr18	10041306	10041320	3.56E-08	AGAACATC	Tier one	Intergenic	VAPA	127358	0	0
chr19	1111261	1111275	3.56E-08	AGAACAGC	Tier one	intron(NM_	GPX4	6619	0	0
chr19	2893817	2893831	3.56E-08	AGAACAGC	Tier one	Intergenic	ZNF57	-7072	0	0
chr2	86055742	86055756	3.56E-08	AGAACAGC	Tier one	Intergenic	AC105053.	13496	0	0
chr3	10361918	10361932	3.56E-08	AGAACACA	Tier one	intron(NM_	SEC13	800	0	0
chr3	15823714	15823728	3.56E-08	AGAACAGA	Tier one	intron(NM_	ANKRD28	15970	0	0
chr3	55367666	55367680	3.56E-08	AGAACAGC	Tier one	Intergenic	WNT5A	147753	0	0
chr3	1.41E+08	1.41E+08	3.56E-08	AGAACAGA	Tier one	intron(NM_	RASA2	-74619	0	0
chr3	1.74E+08	1.74E+08	3.56E-08	AGAACAGA	Tier one	Intergenic	NAALADL2	-183744	1	1
chr6	49366161	49366175	3.56E-08	AGAACAAC	Tier one	Intergenic	MUT	64873	0	0
chr6	1.38E+08	1.38E+08	3.56E-08	AGAACAGT	Tier one	Intergenic	TNFAIP3	-159463	0	0
chr7	22372116	22372130	3.56E-08	AGAACATC	Tier one	intron(NM_	RAPGEF5	24410	0	0
chr7	45277849	45277863	3.56E-08	AGAACACC	Tier one	Intergenic	RAMP3	80489	0	0
chr8	51208665	51208679	3.56E-08	AGAACACC	Tier one	intron(NM_	SNTG1	384333	0	0
chr9	96094045	96094059	3.56E-08	AGAACACT	Tier one	intron(NM_	C9orf129	14644	0	0
chr1	1.7E+08	1.7E+08	6.96E-08	AGAACACA	Tier one	Intergenic	LINC01142	-72804	0	0
chr1	1.8E+08	1.8E+08	6.96E-08	AGAACAAC	Tier one	intron(NM_	NPHS2	15455	1	1
chr1	2.02E+08	2.02E+08	6.96E-08	AGAACATA	Tier one	intron(NR_	RNU6-79P	18778	0	0
chr1	2.42E+08	2.42E+08	6.96E-08	AGAACAAC	Tier one	intron(NM_	WDR64	36672	0	0
chr12	557575	557589	6.96E-08	AGAACACT	Tier one	Intergenic	B4GALNT3	-11961	0	0
chr12	19869306	19869320	6.96E-08	AGAACAGA	Tier one	Intergenic	AEBP2	275798	0	0
chr12	28231555	28231569	6.96E-08	AGAACAAC	Tier one	Intergenic	PTHLH	-106646	0	0
chr12	1.31E+08	1.31E+08	6.96E-08	AGAACATG	Tier one	Intergenic	RIMBP2	-43036	0	0
chr13	50152929	50152943	6.96E-08	AGAACAAA	Tier one	intron(NM_	RCBTB1	6783	0	0
chr13	85331104	85331118	6.96E-08	AGAACATT	Tier one	Intergenic	LINC00351	-606627	0	0
chr15	62592903	62592917	6.96E-08	AGAACAGT	Tier one	Intergenic	MIR8067	4023	0	0
chr16	65331571	65331585	6.96E-08	AGAACAGA	Tier one	intron(NR_	CDH11	-175659	0	0
chr18	41408976	41408990	6.96E-08	AGAACACT	Tier one	Intergenic	SYT4	-551368	1	1
chr2	7669159	7669173	6.96E-08	AGAACACA	Tier one	Intergenic	LOC10050€	107774	0	0
chr2	2.03E+08	2.03E+08	6.96E-08	AGAACAAG	Tier one	exon (NM_	FAM117B	-78774	0	0
chr22	24030343	24030357	6.96E-08	AGAACAAC	Tier one	intron(NR_	RGL4	-2698	0	0
chr4	1.46E+08	1.46E+08	6.96E-08	AGAACAGA	Tier one	Intergenic	SMAD1	-138243	0	0
chr4	1.62E+08	1.62E+08	6.96E-08	AGAACATG	Tier one	Intergenic	FSTL5	1408020	0	0
chr5	14842975	14842989	6.96E-08	AGAACACA	Tier one	intron(NM_	MIR4637	-16861	0	0

chr5	16824092	16824106	6.96E-08	AGAACAGT	Tier one	intron(NM_	MYO10	112286	1	1
chr6	55685964	55685978	6.96E-08	AGAACATG	Tier one	intron(NM_	BMP5	54404	0	0
chr7	77662967	77662981	6.96E-08	AGAACAAT	Tier one	intron(NM_	PHTF2	193527	0	0
chr7	1.47E+08	1.47E+08	6.96E-08	AGAACACA	Tier one	intron(NR_	MIR548F4	-36946	0	0
chr7	1.52E+08	1.52E+08	6.96E-08	AGAACAGA	Tier one	Intergenic	PRKAG2	-24344	0	0
chr8	55880972	55880986	6.96E-08	AGAACAAT	Tier one	Intergenic	XKR4	-134038	0	0
chr8	1.1E+08	1.1E+08	6.96E-08	AGAACAAA	Tier one	Intergenic	TRHR	-73446	0	0
chr8	1.23E+08	1.23E+08	6.96E-08	AGAACATC	Tier one	Intergenic	HAS2	-439372	0	0
chr8	1.27E+08	1.27E+08	6.96E-08	AGAACAGA	Tier one	Intergenic	LINC00861	-178377	0	0
chr9	12065217	12065231	6.96E-08	AGAACACT	Tier one	Intergenic	TYRP1	-628162	0	0
chr9	1E+08	1E+08	6.96E-08	AGAACAAT	Tier one	intron(NM_	NCBP1	-21693	0	0
chr9	1.11E+08	1.11E+08	6.96E-08	AGAACATT	Tier one	Intergenic	ACTL7B	437430	0	0
chr1	1.62E+08	1.62E+08	8.34E-08	AGAACAAT	Tier one	intron(NM_	NOS1AP	-8861	0	0
chr10	53307847	53307861	8.34E-08	AGAACAAT	Tier one	intron(NM_	CSTF2T	151501	1	1
chr11	1.26E+08	1.26E+08	8.34E-08	AGAACATT	Tier one	intron(NM_	CDON	5006	0	0
chr19	19827639	19827653	8.34E-08	AGAACATT	Tier one	intron(NM_	ZNF14	16275	0	0
chr3	1.88E+08	1.88E+08	8.34E-08	AGAACATA	Tier one	intron(NM_	LPP	1118	0	0
chr5	57251906	57251920	8.34E-08	AGAACATT	Tier one	Intergenic	RP11-478P	-56924	0	0
chr5	1.72E+08	1.72E+08	8.34E-08	AGAACATT	Tier one	intron(NM_	CREBRF	8784	0	0
chr8	74072582	74072596	8.34E-08	AGAACAAA	Tier one	Intergenic	SBSPON	-67082	0	0
chr8	82026011	82026025	8.34E-08	AGAACATT	Tier one	Intergenic	PAG1	-1715	0	0
chr8	1.08E+08	1.08E+08	8.34E-08	AGAACAAA	Tier one	Intergenic	ABRA	-155611	0	0
chr8	1.29E+08	1.29E+08	8.34E-08	AGAACAAT	Tier one	Intergenic	MIR1208	7364	0	0
chr9	13677770	13677784	8.34E-08	AGAACAAA	Tier one	Intergenic	FLJ41200	-246449	0	0
chr9	39121844	39121858	8.34E-08	AGAACATA	Tier one	intron(NM_	CNTNAP3	166449	0	0
chr1	17313383	17313397	1.84E-07	AGAACACC	Tier two	exon (NM_	MFAP2	-5309	0	0
chr1	1.53E+08	1.53E+08	1.84E-07	AGCACAGC	Tier two	Intergenic	IVL	4563	0	0
chr1	1.69E+08	1.69E+08	1.84E-07	AGAACAGC	Tier two	Intergenic	XCL1	55359	0	0
chr1	1.76E+08	1.76E+08	1.84E-07	AGAGCAGC	Tier two	intron(NM_	PAPPA2	36806	0	0
chr1	1.79E+08	1.79E+08	1.84E-07	AGAGCACC	Tier two	Intergenic	C1orf220	56950	0	0
chr1	2.07E+08	2.07E+08	1.84E-07	AGCACACC	Tier two	Intergenic	C1orf116	22574	1	-1
chr10	29710421	29710435	1.84E-07	AGACCAGC	Tier two	non-coding	PTCHD3P1	11927	0	0
chr10	44783019	44783033	1.84E-07	AGAGCAGC	Tier two	Intergenic	LOC10013C	-5172	0	0
chr10	61102657	61102671	1.84E-07	AGAGCAGC	Tier two	intron(NM_	FAM13C	19688	0	0
chr10	80037803	80037817	1.84E-07	AGGACAGC	Tier two	intron(NR_	LINC00595	10725	0	0
chr11	62355387	62355401	1.84E-07	AGGACAGC	Tier two	intron(NM_	TUT1	3715	0	0
chr12	71869345	71869359	1.84E-07	AGAGCAGC	Tier two	intron(NM_	LGR5	35802	0	0
chr12	1.24E+08	1.24E+08	1.84E-07	AGAACCCC	Tier two	intron(NM_	CCDC92	9671	0	0
chr12	1.34E+08	1.34E+08	1.84E-07	AGAGCAGC	Tier two	Intergenic	ANHX	23458	0	0
chr13	73663917	73663931	1.84E-07	AGAGCAGC	Tier two	Intergenic	KLF5	30994	0	0
chr13	77962109	77962123	1.84E-07	AGAACAGC	Tier two	Intergenic	MYCBP2	-60939	0	0
chr14	51776667	51776681	1.84E-07	AGAACAGC	Tier two	Intergenic	LINC00640	-23437	0	0
chr14	69078471	69078485	1.84E-07	AGAACAGC	Tier two	Intergenic	ZFP36L1	182153	1	1
chr14	93202453	93202467	1.84E-07	GGAACACC	Tier two	intron(NM_	LGMN	12587	0	0
chr14	1E+08	1E+08	1.84E-07	AGAACAGC	Tier two	Intergenic	EML1	-52374	0	0
chr15	86077547	86077561	1.84E-07	AGAACAGC	Tier two	intron(NM_	AKAP13	-85627	0	0
chr15	99637320	99637334	1.84E-07	AGAACACC	Tier two	Intergenic	SYNM	-7959	0	0

13980 gen	16742 gen	657 genes	685 genes	582 genes	585 genes	102 genes	177 genes
All genes c	All genes c	Genes Posi	Genes Neg	Genes Posi	Genes Neg	Genes Posi	Genes Posi
MIR5690	HS3ST5	LRRC40	ST3GAL1	NAALADL2	C1orf116	CDCA8	LOC150776
MIR592	MIR3660	RRP15	PBX1	NPHS2	CNEP1R1	ARSG	CDC6
LPHN2	MYLK	CDCA7	LTBP1	SYT4	VAMP3	TXNRD1	CENPM
SAMD13	LRRC40	MYC	RP11-115J1	MYO10	IER5	ZNF529	LRP8
PARD3-AS1	PHKB	WDYHV1	GABRB2	CSTF2T	RPS6KC1	LSS	IL17RB
PDLIM1	TFEC	PPARGC1B	HABP2	ZFP36L1	LUZP2	PLA1A	C9orf142
RCN1	CDH8	LOC15077€	RB1CC1	RARB	ST8SIA1	MCM4	CALU
LRTM2	RRP15	DPYSL3	KCNJ3	CDCA8	DUSP6	GNAI3	IFNAR1
C12orf79	NMUR2	EFNA5	ANO4	DUSP10	SEMA6D	C9orf114	DR1
TEX26	UBE3D	TNS3	NCAM2	TUB	LINC00673	ORC1	EOGT
ARHGAP17	PPP1R27	RHBDD1	SH3RF1	CCDC90B	RALGAPA2	CRELD2	OSGEP
VPS4A	B3GALT2	WWC1	RIMS1	COLCA1	ZPLD1	ADM2	PDK1
VAPA	FAR2	UBXN2B	DSC2	USP43	SLITRK3	MCM2	KLK2
GPX4	MIR3147	ASAP1	BPGM	ARSG	AFF1	CETN3	MEIOB
ZNF57	SNORD35A	LINC01003	MTMR9	MAP2K6	SERINC5	MTRNR2L3	MEST
AC105053.	FRMD3	SLCO2A1	ROBO1	GPI	FAM174A	OR5F1	ELSPBP1
SEC13	KCNS2	BMPR1B	HIBADH	CD47	POU3F2	SNX32	MAK
ANKRD28	TAS2R38	HIF1AN	ETV1	CXCL13	EBF2	TREX1	GPX8
WNT5A	ST3GAL1	PTP4A1	C12orf49	EFNA5	SNX16	CDC23	TCOF1
RASA2	FCRL5	ZNF678	IGLL5	FUCA2	FAM154A	HIST1H2BN	NAA38
NAALADL2	PBX1	OSGIN2	AC019118.	MRPS28	NLGN4Y-AS	ZNF280C	RRP9
MUT	FGD3	RFC3	FAM84A	AR	DAB1-AS1	DDIT4	GTPBP4
TNFAIP3	WI2-2373I1	PIGM	PPP1R3B	KCNMA1	CELF3	HES7	SEPT3
RAPGEF5	RP11-364P	GRAMD1C	BRINP3	PIGV	ABL2	CDC45	TRIM35
RAMP3	ETNK1	PTPRZ1	CALCOCO1	LRRC8B	MYOF	BOP1	DHRS13
SNTG1	RACGAP1P	TMEM241	PTPN14	F3	PGM2L1	IFNA5	CMC2
C9orf129	MN1	ADAT2	FAM83B	PC	NTN4	ZNF485	API5
LINC01142	CHRM3	FAM136A	PPP2R5A	NT5DC3	ANO4	HN1	EPHX4
NPHS2	ZNF695	UCHL5	GLI3	TXNRD1	DYNLL1-AS	PAIP2B	PYCR1
RNU6-79P	KCNU1	ERVMER34	ARHGEF3	LOC72873S	PRKD1	POLA1	ZNF559
WDR64	MIR1973	AVPR1A	RP11-290F	PLEK2	COCH	TIMM21	HSPA2
B4GALNT3	GAN	ADARB1	LIPH	PER1	SAMD15	PGGT1B	GHR
AEBP2	MIR549	FAM46C	TTBK2	LINC00669	LAMA3	NOP16	SIRT5
PTHLH	C1orf192	KCNMA1	RAPGEF2	ZNF529	ASXL3	CEP57L1	PRPS2
RIMBP2	MIR1275	YAE1D1	STXBP5L	LSS	FLRT3	ZNF789	MARC1
RCBTB1	KCNIP4-IT1	AKAP1	PTPRR	TMEM39A	FOXP1	VGF	KAT2A
LINC00351	C6orf58	CDC6	LUZP2	SPOCK1	EVC2	HSD17B10	TMEM171
MIR8067	LTBP1	CYP2C8	CXADR	PTP4A1	UGT2B10	ACOT2	EFEMP1
CDH11	GALP	LRRC59	CDC14C	MPP6	TSLP	MTIF2	ERCC6L
SYT4	LOC28608:	PRIM1	NCOA2	TNS3	KIF13B	LAMP3	CHAC1
LOC10050€	KIFAP3	PNPT1	MEG9	TMEM243	SERPINH1	ERMARD	TDRD9
FAM117B	RP11-115J1	ADRA2A	ARHGAP32	PTPRZ1	ROBO1	CA3	WNT10B
RGL4	LOC10260€	RPSAP58	DOK6	GRIN3A	FSTL1	MTERFD1	DDIT3
SMAD1	CYP26B1	ALG6	MET	AVPR1A	PDE7B	TMEM41B	POGLUT1
FSTL5	CDCA7	LINC00624	ASXL3	PHLDB2	MTSS1	E2F5	SSFA2

MIR4637	NPBWR1	CD47	TNFSF15	CTH	PBX1	KATNAL2	GNG12
MYO10	LINC00706	ZMIZ1	MGAT4C	C1orf112	C11orf70	DHODH	ALKBH2
BMP5	PER4	DIRC3	ATG14	RHOU	TNRC6A	MRPL17	CNTD2
PHTF2	MYC	TOM1L1	EPHA6	DLG2	PCTP	WDR76	PIBF1
MIR548F4	WDYHV1	ELK4	AMIGO2	SELT	BCAS3	ZP3	FAM135A
PRKAG2	RNF219-AS	SLC35B4	INADL	PLEKHG1	BTG3	DIMT1	TLR3
XKR4	SMARCA2	MRPS28	LHX9	JPH1	LRRN1	HAPLN3	LAT2
TRHR	DCC	LOC10012	PTPN12	RRP15	KAT2B	SLC7A5	NSMCE1
HAS2	WDR1	PLEKHG1	RCBTB2	SESN3	THRB	CCDC109B	MYBBP1A
LINC00861	KDM4E	PCYOX1L	RORB	ALG10	CADM2	AKIRIN1	NOP56
TYRP1	CYP2R1	DSN1	ARHGAP35	TMEM5	AREG	ZFP69B	NTAN1
NCBP1	PPARGC1B	CENPM	MDGA1	DHRS2	TET2	PCGF6	KIF15
ACTL7B	LOC10192	LRP8	AK8	IRX3	CTNND2	MAGOHB	DHRS11
NOS1AP	VSTM2B	SLC38A4	EPHA7	MAFB	TWIST1	SMAGP	OXNAD1
CSTF2T	GLP1R	SLC39A14	EFCAB6	BMPR1B	CDC14C	UNG	CSTF2
CDON	FZD2	DLG2	PDE7B	TMEM14A	ATXN7L3B	VPS9D1-AS	GGNBP2
ZNF14	GNAQ	SYT4	SEMA6D	FAM91A1	CDADC1	SARM1	RPF2
LPP	RSL24D1	IL17RB	SPOCK3	RGS2	SPOCK3	PNPO	WDR62
RP11-478P	CAMK1D	HNRNPD	UGT2B10	OTUD1	FAM65B	TYMS	PUS1
CREBRF	MALSU1	IRX3	UNC5C	PLA1A	PHIP	ABHD3	PLA2G4F
SBSPON	NCOA7	AKAP12	ALB	MCM4	GPR126	CYB5A	TMED2
PAG1	HSPA12B	C9orf142	GC	NR2F2	LMTK2	ILF3-AS1	SLAIN1
ABRA	LBR	NAALADL2	DSP	GNAI3	GOLGA7	PIH1D1	SLC1A4
MIR1208	XXYLT1-AS	CIRH1A	CTTNBP2N	HSD17B7	TRPS1	NIF3L1	ITPRIPL2
FLJ41200	RP11-1C1.4	SLC29A1	BMP2	TSEN15	USP54	METTL21A	TP53
CNTNAP3	ESRG	CTSL	ITM2C	GLRX3	TMEM47	SDF2L1	ZNF420
MFAP2	MED15	ZFP36L1	ZBBX	FAR1	DENND1B	FANCD2	CPSF2
IVL	HSPA9	BHLHA15	STK38L	MMAB	BICC1	FAM86JP	C1orf74
XCL1	DYSF	CCKBR	IQCG	NLK	LIMA1	HIST1H4D	SFXN2
PAPPA2	HES1	PHLDB2	SLC35D1	SEH1L	CPM	IFITM4P	NUS1
C1orf220	OR6W1P	PTPLB	GRM8	HEG1	NTS	PSPH	RECQL
C1orf116	GABRB2	CALU	TNFRSF21	YTHDC2	MKL2	PSMC2	BAG2
PTCHD3P1	ACTR3B	RPIA	CHRM2	AKAP12	CDH2	MYBL1	CXorf57
LOC10013	MKRN9P	THBS1	ANKRD22	PDK4	SATB2	TONSL	DENND4A
FAM13C	MKRN2	HDGFRP3	CDH2	SLC25A37	KIF16B	FUNDC1	MTTP
LINC00595	PRSS35	PLEK2	CTNND2	POP1	RFTN1	RBMY2EP	CNOT6L
TUT1	PCDH18	LINC00669	VAMP3	C9orf114	SI	SLC47A1	NUFIP1
LGR5	RAP2A	SOD2	REEP3	ORC1	WDFY3	CCDC58	TRIM59
CCDC92	RNF182	SLC45A3	ACER2	DAB1	DSP	MXRA8	IL6ST
ANHX	RP11-71G7	IFNAR1	IQGAP1	LRRC40	IRAK1BP1	CITED4	CD320
KLF5	RNF217	CXCL13	MXRA7	LINC00624	SEMA3C	TOE1	ZDHHC23
MYCBP2	NLRC3	ADPGK	MVB12B	XPR1	PPP2R2A	HIST2H2AB	SLC15A2
LINC00640	ETV3	RARB	AGAP11	C1orf21	UHMK1	LINC00959	ZNF582
ZFP36L1	WI2-81516	FAM84B	INTU	ZNF678	PPP1R12A	SHMT2	C6orf211
LGMN	ZFYVE21	TXNDC16	FAM149B1	GPR137B	KCNJ3	CHRNA5	ATL3
EML1	AGL	EXO1	EPHA2	EXO1	SLCO5A1	ZNF551	RP11-314C16.1
AKAP13	TMEM135	NKX3-1	C10orf12	PPIF	BRINP3	GALM	TIMM13

480 genes	58 genes	158 genes	527 genes	368 genes	360 genes	33 genes	50 genes
Genes Posi	Genes Neg	Genes Neg	Genes Neg	KLF respon	KLF respon	KLF respon	KLF respon
NAALADL2	PGM2L1	DSC2	C1orf116	MXRA8	SKI	PIGV	INADL
NPHS2	DYNLL1-AS	ATG14	CNEP1R1	TMEM52	TPRG1L	CHD1L	TUFT1
SYT4	SAMD15	RCBTB2	VAMP3	PIGV	TP73	LINC00624	PBX1
MYO10	THRB	ALB	IER5	RCC1	VAMP3	RGS4	IER5
CSTF2T	KIAA1467	ITM2C	RPS6KC1	MECR	CASZ1	FAM129A	BRINP3
ZFP36L1	JUN	IQCG	LUZP2	CDCA8	EPHA2	ZNF678	LUZP2
RARB	KIAA1324	ANKRD22	ST8SIA1	HPDL	CELA3B	RHOU	SYTL2
DUSP10	ELF3	IQGAP1	DUSP6	TOE1	RCAN3	ARL5B	CWF19L2
TUB	DZIP1	AGAP11	SEMA6D	ORC1	EFCAB14	PPIF	SLC16A7
CCDC90B	MARK4	FAM149B1	LINC00673	DAB1	SPATA6	GLRX3	CPM
COLCA1	DOCK10	LZTFL1	RALGAPA2	ALG6	JUN	FTH1	KIAA1033
USP43	GSTA2	TMEM133	ZPLD1	CTH	INADL	SESN3	C12orf49
MAP2K6	SLC35D2	ZG16B	SLITRK3	LPAR3	RAVER2	STYK1	ATP12A
GPI	GAS2	BHLHA9	AFF1	LRRC8B	NTNG1	ALG10	SLITRK6
CD47	VRK3	RP11-84O1	SERINC5	F3	KIAA1324	IGF1	DICER1
CXCL13	TRAF3IP1	RSPH1	FAM174A	CNN3	CTTNBP2N	THBS1	CBLN2
EFNA5	MEAF6	GIT2	POU3F2	LOC10012S	ANKRD20A	EEF2K	SATB2
FUCA2	CLIP1	SOGA3	EBF2	GNAI3	BCL9	AKAP1	RAMP1
MRPS28	RRN3P2	KIAA0825	SNX16	ATP1A1	TUFT1	LINC01029	GAL3ST2
AR	IZUMO2	MROH2A	FAM154A	FAM46C	CELF3	CHAC2	NCOA3
KCNMA1	AMOTL2	GDAP1	NLGN4Y-AS	CHD1L	S100A10	FOXRED2	RBM11
PIGV	SMIM14	PCDH11X	DAB1-AS1	LINC00624	GATAD2B	CD47	LRRN1
LRRC8B	MST4	TEAD3	CELF3	HIST2H2AB	PBXIP1	PTPLB	RFTN1
F3	MOV10	WDR91	ABL2	SV2A	ATP1A2	PTGER4	SATB1
PC	EXPH5	MIR30A	MYOF	PMVK	UHMK1	STC2	NEK10
NT5DC3	ANKRD42	HOXA13	NTN4	PIGM	PBX1	PRIM2	ROBO1
LOC72873S	KCTD13	HLA-DRA	ANO4	HSD17B7	ABL2	TNS3	EPHA6
PLEK2	A1CF	ITLN1	PRKD1	RGS4	SOAT1	XKR6	ABTB1
PER1	SLC10A7	MAPK13	COCH	TNFSF4	IER5	SLC25A37	ZBBX
LINC00669	HLA-DQB1	SYTL5	LAMA3	NPHS2	BRINP3	JPH1	KIAA1211
TMEM39A	SNAP23	LINC00674	ASXL3	XPR1	DENND1B	FAM84B	AREG
SPOCK1	TNKS2	CCDC146	FLRT3	TSEN15	LHX9	ALDH1B1	WDFY3
PTP4A1	ZNF844	UBE4B	FOXP1	C1orf21	ELF3	CNTRL	DDIT4L
MPP6	GSTT1	DMRT3	EVC2	FAM129A	C1orf116		ARSJ
TNS3	TPRG1L	OTX1	UGT2B10	RGS2	SLC30A1		KIAA1109
TMEM243	RNF11	SCAPER	TSLP	UCHL5	PPP2R5A		RAI14
PTPRZ1	TET1	KIAA0513	KIF13B	ELK4	RPS6KC1		NR3C1
GRIN3A	NODAL	ADAMTS1	SERPINH1	SLC45A3	PTPN14		EPHA7
AVPR1A	NUDT4P1	WDR96	ROBO1	SLC41A1	CCSAP		PTPRK
PHLDB2	SLC30A4	CGN	FSTL1	RRP15	GATA3		ETV1
CTH	ERBB2	GABRQ	PDE7B	MARC1	PLXDC2		MKLN1
C1orf112	STAT3	OR2J2	MTSS1	HLX	A1CF		BNIP3L
RHOU	PGPEP1	LGALS8-AS	PBX1	DUSP10	BICC1		GTF2E2
DLG2	ZBTB32	CALML4	C11orf70	TAF1A	REEP3		MMP16
SELT	C2orf50	SGTB	TNRC6A	FBXO28	TET1		TRPS1

PLEKHG1	GPCPD1	TEX9	PCTP	ZNF678	SPOCK2		AK3
JPH1	FAM227A	KRT33B	BCAS3	RHOU	USP54		RORB
RRP15	SLC12A7	ZSWIM4	BTG3	B3GALNT2	HECTD2		TMEM38B
SESN3	ST6GALNA	ERBB4	LRRN1	NID1	MYOF		RP11-40F8.
ALG10	LINC00278	CEP89	KAT2B	EXO1	LCOR		EDA2R
TMEM5	AGO1	DUSP18	CADM2	ST8SIA6	C10orf12		
DHRS2	CASC1	DUSP8	AREG	ARL5B	BTRC		
IRX3	NBR1	UBXN11	TET2	PDSS1	CNNM2		
MAFB	GDF15	RIT1	CTNND2	HSD17B7P	HABP2		
BMPR1B	LYPD3	ANKRD52	TWIST1	ZNF485	FAM160B1		
TMEM14A	C2orf54	HLA-DMB	CDC14C	DDIT4	GPR26		
FAM91A1	BRWD1	RHOBTB1	ATXN7L3B	KCNMA1	SMPD1		
RGS2	SERINC1	LRP10	CDADC1	ZMIZ1	KRT8P41		
OTUD1		DNALI1	SPOCK3	PPIF	USP47		
NR2F2		SNCA	FAM65B	HELLS	TEAD1		
HSD17B7		TESK2	PHIP	TLL2	ARNTL		
TSEN15		NIN	GPR126	HIF1AN	GAS2		
GLRX3		KCNE3	LMTK2	NOLC1	LUZP2		
FAR1		RAB30	GOLGA7	TRIM8	CCDC34		
MMAB		ERO1L	TRPS1	SFR1	LGR4		
NLK		LRRC31	USP54	ADRA2A	SERPINH1		
SEH1L		TMED8	TMEM47	HTRA1	SYTL2		
HEG1		PSMB9	DENND1B	LINC00959	PRSS23		
YTHDC2		SYP	BICC1	GLRX3	C11orf70		
AKAP12		RP11-40F8	LIMA1	TUB	CWF19L2		
PDK4		CPEB2	CPM	FAR1	EXPH5		
SLC25A37		HMGCS2	NTS	GLYATL2	BCO2		
POP1		ZNF217	MKL2	FTH1	ARHGAP32		
DAB1		RAB9A	CDH2	SNX32	CLSTN3		
LRRC40		OCLN	SATB2	SLC29A2	DUSP16		
LINC00624		CLEC18B	KIF16B	PC	CREBL2		
XPR1		FRAT2	RFTN1	TSKU	GPRC5A		
C1orf21		LINC01136	SI	CCDC90B	ST8SIA1		
ZNF678		FAM183A	WDFY3	DLG2	STK38L		
GPR137B		MLLT3	DSP	SESN3	CPNE8		
EXO1		RAB19	IRAK1BP1	COLCA1	ANO6		
PPIF		YPEL2	SEMA3C	ZBTB16	PCED1B		
HIF1AN		DMTN	PPP2R2A	CHEK1	LIMA1		
TSKU		OSCP1	UHMK1	BCAT1	DIP2B		
ZBTB16		CDKN1B	PPP1R12A	ALG10	CALCOCO1		
SLC38A4		WDR44	KCNJ3	SLC38A4	ZNF385A		
IGF1		DIRC2	SLCO5A1	VDR	ITGA5		
WSB2		MIR4686	BRINP3	ASIC1	CTDSP2		
RFC3		NPTN	ARHGAP32	SMAGP	SLC16A7		
THBS1		FBXO8	IFT81	PRIM1	GNS		
NEDD4L		SPINK1	MAP4K4	MYO1A	CPM		
NFIC		PCGF3	ABTB1	SHMT2	PTPRR		

Chr arm	CWR22Rv1	Genes	Amp arm fr	Amp arm q	Del arm fre	Del arm q-value
1p	1.3	1300	0.02	1.0E+00	0.05	1.1E-03
1q	2.6	1195	0.05	5.4E-02	0.02	1.0E+00
2p	1.0	624	0.02	1.0E+00	0.03	1.0E+00
2q	1.0	967	0.01	1.0E+00	0.04	1.0E+00
3p	1.6	644	0.08	3.0E-03	0.02	1.0E+00
3q	1.6	733	0.11	1.7E-10	0.01	1.0E+00
4p	1.0	289	0.02	1.0E+00	0.05	1.0E+00
4q	1.0	670	0.02	1.0E+00	0.03	1.0E+00
5p	1.2	183	0.04	1.0E+00	0.03	1.0E+00
5q	1.2	905	0.03	1.0E+00	0.05	4.8E-01
6p	1.3	710	0.02	1.0E+00	0.06	4.5E-01
6q	1.3	556	0.01	1.0E+00	0.09	1.9E-03
7p	2.0	389	0.21	0.0E+00	0.00	1.0E+00
7q	2.0	783	0.19	0.0E+00	0.01	1.0E+00
8p	2.1	338	0.13	5.6E-07	0.44	0.0E+00
8q	2.1	551	0.25	0.0E+00	0.08	5.4E-02
9p	1.1	301	0.07	1.0E+00	0.05	1.0E+00
9q	1.1	700	0.10	5.6E-07	0.02	1.0E+00
10p	1.3	253	0.03	1.0E+00	0.09	5.9E-02
10q	1.3	738	0.03	1.0E+00	0.09	3.9E-06
11p	1.3	509	0.05	1.0E+00	0.02	1.0E+00
11q	1.3	975	0.06	4.8E-02	0.01	1.0E+00
12p	2.1	339	0.03	1.0E+00	0.10	2.8E-04
12q	2.1	904	0.04	1.0E+00	0.04	8.7E-01
13p	0.9		0.00	1.0E+00	0.00	1.0E+00
13q	0.9	560	0.02	1.0E+00	0.16	0.0E+00
14p	1.0		0.00	1.0E+00	0.00	1.0E+00
14q	1.0	938	0.02	1.0E+00	0.05	1.5E-01
15p	1.0		0.00	1.0E+00	0.00	1.0E+00
15q	1.0	810	0.01	1.0E+00	0.07	5.3E-03
16p	1.3	559	0.07	4.9E-01	0.07	9.4E-02
16q	1.3	455	0.02	1.0E+00	0.22	0.0E+00
17p	1.4	415	0.02	1.0E+00	0.16	0.0E+00
17q	1.4	972	0.02	1.0E+00	0.04	6.0E-01
18p	1.2	104	0.04	1.0E+00	0.14	3.3E-10
18q	1.2	275	0.03	1.0E+00	0.21	0.0E+00
19p	1.4	681	0.02	1.0E+00	0.04	1.0E+00
19q	1.4	935	0.02	1.0E+00	0.04	9.6E-01
20p	1.5	234	0.05	1.0E+00	0.05	1.0E+00
20q	1.5	448	0.06	1.0E+00	0.02	1.0E+00
21p	0.9		0.00	1.0E+00	0.00	1.0E+00
21q	0.9	258	0.04	1.0E+00	0.05	1.0E+00
22p	1.1		0.00	1.0E+00	0.00	1.0E+00
22q	1.1	564	0.01	1.0E+00	0.10	4.7E-05
Xp	0.4	418	0.02	1.0E+00	0.08	6.7E-02
Xq	0.4	668	0.01	1.0E+00	0.08	1.6E-03

Amp focal (	Amp focal (	Amp focal v	Del focal cy	Del focal q	Del focal wide peak boundaries
	1.0E+00		1p22.1	6.1E-03	chr1:82457075-107602611
	1.0E+00		1p31.3	6.4E-04	chr1:63901623-66226788
	1.0E+00		2p21	9.6E-02	chr2:31802826-47133054
	1.0E+00		2q22.1	4.5E-21	chr2:132506741-141000702
	1.0E+00		3p13	1.6E-21	chr3:71874461-72622531
3q21.3	3.1E-05	chr3:12787	3q29	1.8E-01	chr3:194988874-198022430
4p16.3	1.1E-01	chr4:1-2082008		1.0E+00	
4q13.3	8.5E-03	chr4:73892	4q28.2	2.7E-03	chr4:115720296-134070791
5q35.3	1.6E-01	chr5:12067	5q11.2	3.1E-28	chr5:54597198-57789060
	1.0E+00		5q13.2	2.1E-26	chr5:66492413-72750050
	1.0E+00		6p25.1	5.9E-02	chr6:4071224-9597208
	1.0E+00		6q14.3	4.6E-73	chr6:87800060-88001063
7p14.3	2.8E-02	chr7:15167171-38501		1.0E+00	
7q22.1	1.3E-02	chr7:84702970-10868		1.0E+00	
	1.0E+00		8p21.3	5.9E-30	chr8:20153951-24300351
8q22.1	3.7E-05	chr8:64514471-11457		1.0E+00	
	1.0E+00		9p23	1.9E-04	chr9:7887952-12687261
9q33.3	5.9E-02	chr9:101892280-1323		1.0E+00	
	1.0E+00			1.0E+00	
	1.0E+00		10q23.31	6.1E-97	chr10:89617158-90034038
	1.0E+00		11p11.2	5.0E-03	chr11:37721288-49054288
	1.0E+00		11q22.3	2.6E-04	chr11:102955092-122320965
	1.0E+00		12p13.1	4.8E-22	chr12:12844234-12921132
12q23.1	1.6E-02	chr12:761	12q24.32	2.4E-02	chr12:123752264-133851895
	1.0E+00			1.0E+00	
13q12.11	4.7E-02	chr13:1955	13q14.13	1.1E-73	chr13:44451821-48879660
	1.0E+00			1.0E+00	
	1.0E+00			1.0E+00	
	1.0E+00			1.0E+00	
15q11.2	1.5E-02	chr15:1-26	15q21.3	4.2E-02	chr15:56756286-58245985
	1.0E+00			1.0E+00	
16q11.2	2.8E-03	chr16:465	16q22.3	9.0E-33	chr16:72142073-74472459
17p11.2	2.0E-01	chr17:188	17p13.1	1.3E-34	chr17:7505228-7591760
17q23.2	4.0E-03	chr17:597	17q21.31	6.2E-15	chr17:42102307-42651945
	1.0E+00			1.0E+00	
18q21.31	2.1E-01	chr18:559	18q21.31	1.1E-03	chr18:51910315-71741815
	1.0E+00			1.0E+00	
19q13.43	1.8E-01	chr19:538	19q13.2	1.7E-03	chr19:42731571-42891571
	1.0E+00			1.0E+00	
20q13.2	7.1E-02	chr20:41749332-6302		1.0E+00	
	1.0E+00			1.0E+00	
	1.0E+00		21q22.2	1.4E-41	chr21:39665722-40155078
	1.0E+00			1.0E+00	
	1.0E+00			1.0E+00	
	1.0E+00			1.0E+00	
Xq12	2.0E-02	chrX:64162405-67316		1.0E+00	

Xq21.1	2.7E-02	chrX:80829747-80833679
Xq21.31	4.7E-02	chrX:87704771-87710744

Geneset enrichment analysis

Input data and parameters:

User data: ChIPSeq_AR.txt
Organism: hsapiens
Id Type: gene_symbol
Ref Set: entrezgene
Significance Level: 0.05
Statistics Test: Hypergeometric
MTC: BH
Minimum: 2

Output data and parameters:

For each enriched gene set, the first row lists database name, gene set name , and corresponding gene set ID.

Statistics:

C: the number of reference genes in the category
O: the number of genes in the gene set and also in the category
E: the expected number in the category
R: ratio of enrichment
rawP: p value from hypergeometric test
adjP: p value adjusted by the multiple test adjustment
Finally, genes in the pathway are listed. For each gene, the table lists the user uploaded ID and value, Entrez ID, Gene symbol, and description.

WEB-based GEne SeT AnaLysis To
Translating gene lists into biologic

Genes Positively regulate	657		
**PathwayNameKEGG**	**#Gene**	**EntrezGene**	**Statistics**
Metabolic pathways	60	84706 23649 33	C=1130;O=60
DNA replication	8	23649 5983 555	C=36;O=8;E=(
Mismatch repair	6	9156 5983 5982	C=23;O=6;E=(
Ribosome biogenesis in e	9	10556 10940 75	C=80;O=9;E=
Steroid biosynthesis	5	6713 51478 171	C=19;O=5;E=(
Pathways in cancer	16	5915 836 4824 7	C=326;O=16;
Homologous recombinati	5	641 5888 6119 5	C=28;O=5;E=(
Terpenoid backbone bios	4	3157 10654 315	C=15;O=4;E=(
p53 signaling pathway	7	55240 1111 836	C=68;O=7;E=
Pyrimidine metabolism	8	790 23649 8417	C=99;O=8;E=
Arginine and proline meta	6	4128 1152 2992	C=54;O=6;E=(
Purine metabolism	10	23649 5558 486	C=162;O=10;
Protein processing in end	10	91319 64215 62	C=165;O=10;
Peroxisome	7	10654 84188 20	C=79;O=7;E=
Proximal tubule bicarbon	4	476 8671 478 14	C=23;O=4;E=(
Glycolysis / Gluconeogen	6	83440 2821 219	C=65;O=6;E=(
RNA transport	9	10556 10940 75	C=151;O=9;E
Pentose phosphate pathv	4	2821 5211 5634	C=27;O=4;E=(
Glycerolipid metabolism	5	4023 219 12964	C=50;O=5;E=(
Toxoplasmosis	8	10105 836 5163	C=132;O=8;E
Alanine, aspartate and glu	4	790 84706 440 5	C=32;O=4;E=(
Protein digestion and abs	6	476 6520 6510 4	C=81;O=6;E=
Fructose and mannose m	4	9107 8776 5211	C=36;O=4;E=(
Prostate cancer	6	367 354 4824 34	C=89;O=6;E=
MAPK signaling pathway	11	2005 55970 836	C=268;O=11;
Cell cycle	7	27127 1111 990	C=124;O=7;E
Tryptophan metabolism	4	4128 219 51166	C=42;O=4;E=(
Aldosterone-regulated so	4	23327 476 478 3	C=42;O=4;E=(
RNA degradation	5	51691 23404 24	C=71;O=5;E=
Valine, leucine and isoleu	4	586 3157 219 22	C=44;O=4;E=(
Fatty acid metabolism	4	33 219 224 2181	C=43;O=4;E=(
Nucleotide excision repai	4	5983 5982 6119	C=44;O=4;E=(
Pancreatic secretion	6	1811 476 8671 3	C=101;O=6;E
Nitrogen metabolism	3	440 377677 149	C=23;O=3;E=(
N-Glycan biosynthesis	4	29880 29929 91	C=49;O=4;E=(
Neuroactive ligand-recep	10	7433 887 552 26	C=272;O=10;
Basal cell carcinoma	4	5727 7480 8321	C=55;O=4;E=(
Histidine metabolism	3	4128 219 224	C=29;O=3;E=(
Valine, leucine and isoleu	2	586 57176	C=11;O=2;E=(
Pentose and glucuronate	3	219 51181 224	C=32;O=3;E=(
Colorectal cancer	4	836 4609 2956 7	C=62;O=4;E=(

Genes Positively regulate	584		
**PathwayNameKEGG**	**#Gene**	**EntrezGene**	**Statistics**
Metabolic pathways	59	84706 33 93183	C=1130;O=59
DNA replication	8	4173 5983 5558	C=36;O=8;E=(
Steroid biosynthesis	6	6713 4047 5147	C=19;O=6;E=(
Cell cycle	10	8318 4173 4609	C=124;O=10;I
Pyrimidine metabolism	9	7296 5558 4860	C=99;O=9;E=1
Terpenoid backbone bios	4	3157 10654 315	C=15;O=4;E=(
One carbon pool by folate	4	7298 6472 1604	C=18;O=4;E=(
Peroxisome	7	10654 84188 20	C=79;O=7;E=1
Mismatch repair	4	9156 5983 6119	C=23;O=4;E=(
Glycolysis / Gluconeogene	6	83440 2821 130	C=65;O=6;E=(
Nitrogen metabolism	4	440 761 377677	C=23;O=4;E=(
Valine, leucine and isoleu	5	586 3157 219 22	C=44;O=5;E=(
Glycerolipid metabolism	5	4023 219 12964	C=50;O=5;E=(
Homologous recombinati	4	641 5888 6119 2	C=28;O=4;E=(
Protein digestion and abs	6	476 6520 6510 4	C=81;O=6;E=1
Arginine and proline meta	5	4128 1152 2992	C=54;O=5;E=(
Glycine, serine and threo	4	4128 6472 5723	C=32;O=4;E=(
Selenocompound metabo	3	7296 92935 149	C=17;O=3;E=(
Pathways in cancer	12	5915 4824 3480	C=326;O=12;I
p53 signaling pathway	5	55240 1111 143	C=68;O=5;E=(
Tryptophan metabolism	4	4128 219 51166	C=42;O=4;E=(
Pancreatic secretion	6	1811 476 8671 3	C=101;O=6;E=
Aldosterone-regulated so	4	23327 476 478 3	C=42;O=4;E=(
Fatty acid metabolism	4	33 219 224 2181	C=43;O=4;E=(
Biosynthesis of unsaturat	3	60481 10965 20	C=21;O=3;E=(
Proximal tubule bicarbon	3	476 8671 478	C=23;O=3;E=(
RNA transport	7	79833 10940 55	C=151;O=7;E=
Purine metabolism	7	84172 5558 486	C=162;O=7;E=
Histidine metabolism	3	4128 219 224	C=29;O=3;E=(
Pentose and glucuronate	3	219 51181 224	C=32;O=3;E=(
Toxoplasmosis	6	10105 345611 3	C=132;O=6;E=
Alanine, aspartate and glu	3	84706 440 5471	C=32;O=3;E=(
Neuroactive ligand-recep	9	7433 1138 552 8	C=272;O=9;E=
Fructose and mannose m	3	9107 8776 3099	C=36;O=3;E=(
Long-term depression	4	3480 2773 5048	C=70;O=4;E=(
Bile secretion	4	476 8671 478 31	C=71;O=4;E=(
Pyruvate metabolism	3	5091 219 224	C=40;O=3;E=(
Gastric acid secretion	4	476 2773 887 47	C=74;O=4;E=(
Oocyte meiosis	5	27127 367 8697	C=112;O=5;E=

Genes Negatively regulated by ARE half sites

PathwayNameKEGG	#Gene	EntrezGene	Statistics
Axon guidance	16	1948 8633 2852	C=129;O=16;
ErbB signaling pathway	10	27 6655 374 844	C=87;O=10;E=
Focal adhesion	12	10398 6655 390	C=200;O=12;
Bacterial invasion of epith	7	3678 4233 5291	C=70;O=7;E=
Tight junction	9	9076 9414 1039	C=132;O=9;E=
Cell adhesion molecules (	9	9076 3122 4685	C=133;O=9;E=
Protein digestion and abs	7	10008 1803 481	C=81;O=7;E=
Sphingolipid metabolism	5	166929 55304 3	C=40;O=5;E=
Regulation of actin cytosk	11	10398 6655 293	C=213;O=11;
p53 signaling pathway	6	7161 900 56475	C=68;O=6;E=
Neurotrophin signaling pa	8	7161 6655 5749	C=127;O=8;E=
Adherens junction	6	1387 51176 423	C=73;O=6;E=
Mucin type O-Glycan bios	4	2591 51301 555	C=30;O=4;E=
Insulin signaling pathway	8	79660 2872 665	C=138;O=8;E=
Pathogenic Escherichia co	5	9076 6093 8976	C=56;O=5;E=
TGF-beta signaling pathw	6	650 1387 4052 3	C=84;O=6;E=
Leukocyte transendotheli	7	9076 10398 560	C=116;O=7;E=
Shigellosis	5	3678 5603 6093	C=61;O=5;E=
Toxoplasmosis	7	3122 5603 3909	C=132;O=7;E=
Renal cell carcinoma	5	1387 6655 4233	C=70;O=5;E=
Hepatitis C	7	9076 5520 6655	C=134;O=7;E=
Carbohydrate digestion a	4	481 5291 477 64	C=44;O=4;E=
Pancreatic secretion	6	481 5874 23436	C=101;O=6;E=
Circadian rhythm - mamm	3	6096 8945 406	C=22;O=3;E=
Chronic myeloid leukemia	5	1027 6655 5291	C=73;O=5;E=
Arrhythmogenic right ven	5	3678 51176 182	C=74;O=5;E=
Wnt signaling pathway	7	23401 1387 609	C=150;O=7;E=
MAPK signaling pathway	10	6655 1848 1850	C=268;O=10;
ECM-receptor interaction	5	3678 3909 1290	C=85;O=5;E=
Salivary secretion	5	5593 481 477 49	C=89;O=5;E=
Hematopoietic cell lineag	5	3678 966 3122 1	C=88;O=5;E=
Prostate cancer	5	1027 1387 6655	C=89;O=5;E=
Pathways in cancer	11	650 1387 6655 2	C=326;O=11;

Genes Negatively regulated by ARE full sites

PathwayNameKEGG	#Gene	EntrezGene	Statistics
Axon guidance	15	1948 8633 2852	C=129;O=15;
Focal adhesion	13	3725 10398 665	C=200;O=13;
ErbB signaling pathway	9	3725 27 6655 37	C=87;O=9;E=
Renal cell carcinoma	6	3725 1387 6655	C=70;O=6;E=
Sphingolipid metabolism	5	166929 55304 3	C=40;O=5;E=
Bacterial invasion of epith	6	3678 4233 5291	C=70;O=6;E=
p53 signaling pathway	6	7161 900 56475	C=68;O=6;E=
Adherens junction	6	2064 1387 5117	C=73;O=6;E=
Regulation of actin cytosk	10	10398 6655 293	C=213;O=10;
Pathways in cancer	13	3725 650 1387 6	C=326;O=13;
Mucin type O-Glycan bios	4	2591 51301 555	C=30;O=4;E=
Protein digestion and abs	6	1803 481 1290 2	C=81;O=6;E=
Glycosphingolipid biosynt	3	6489 6482 3081	C=15;O=3;E=
TGF-beta signaling pathw	6	650 1387 4052 6	C=84;O=6;E=
Neurotrophin signaling pa	7	3725 7161 6655	C=127;O=7;E
Cell adhesion molecules (	7	9076 1000 4685	C=133;O=7;E
Pancreatic secretion	6	481 5874 23436	C=101;O=6;E
Insulin signaling pathway	7	79660 2872 665	C=138;O=7;E
Carbohydrate digestion a	4	481 5291 477 64	C=44;O=4;E=
Circadian rhythm - mamm	3	6096 8945 406	C=22;O=3;E=
Wnt signaling pathway	7	3725 1387 6093	C=150;O=7;E
Endometrial cancer	4	2064 6655 5117	C=52;O=4;E=
ECM-receptor interaction	5	3678 3909 1290	C=85;O=5;E=
Prostate cancer	5	2064 1387 6655	C=89;O=5;E=
Salivary secretion	5	5593 481 477 49	C=89;O=5;E=
Pathogenic Escherichia co	4	9076 6093 8976	C=56;O=4;E=
Acute myeloid leukemia	4	6655 51176 677	C=57;O=4;E=
Hepatitis C	6	9076 5520 6655	C=134;O=6;E
Shigellosis	4	3678 6093 8976	C=61;O=4;E=
Tight junction	6	9076 5520 9414	C=132;O=6;E
MAPK signaling pathway	9	3725 6655 1848	C=268;O=9;E
Glycosphingolipid biosynt	2	6489 6482	C=14;O=2;E=
Metabolism of xenobiotic	4	2952 2941 2939	C=71;O=4;E=
T cell receptor signaling p	5	3725 6655 5291	C=108;O=5;E
RNA degradation	4	10950 54464 78	C=71;O=4;E=
Amoebiasis	5	3909 1290 5291	C=106;O=5;E
Metabolic pathways	24	1788 669 30815	C=1130;O=24
Arrhythmogenic right ven	4	3678 51176 183	C=74;O=4;E=
Drug metabolism - cytoch	4	2952 2941 2939	C=73;O=4;E=
Jak-STAT signaling pathwa	6	1387 6655 6774	C=155;O=6;E
Aldosterone-regulated so	3	481 5291 477	C=42;O=3;E=
Vascular smooth muscle c	5	10398 6093 102	C=116;O=5;E
Leukocyte transendotheli	5	9076 10398 609	C=116;O=5;E

KLF response eleme	368		
**PathwayName**	**#Gene**	**EntrezGene**	**Statistics**
Metabolic pathway:	39	84706 93183 73	C=1130;O=39
Peroxisome	6	10654 2053 841	C=79;O=6;E=(
One carbon pool by	3	6472 160428 45	C=18;O=3;E=(
Terpenoid backbon	3	10654 3156 235	C=15;O=3;E=(
Aldosterone-regula	4	23327 476 478 3	C=42;O=4;E=(
p53 signaling pathw	5	1111 55240 143	C=68;O=5;E=(
Cell cycle	6	27127 1111 499	C=124;O=6;E=
Nitrogen metabolis	3	440 761 1491	C=23;O=3;E=(
Pancreatic secretio	5	1811 476 3778 5	C=101;O=5;E=
Pathways in cancer	9	5915 4824 3480	C=326;O=9;E=
Glycine, serine and	3	4128 6472 1491	C=32;O=3;E=(
Long-term depressi	4	3480 2773 5048	C=70;O=4;E=(
DNA replication	3	5983 5558 5557	C=36;O=3;E=(
Tryptophan metabc	3	4128 219 51166	C=42;O=3;E=(
Progesterone-medi	4	3480 8697 2773	C=86;O=4;E=(
Toxoplasmosis	5	10105 3459 277	C=132;O=5;E=
Valine, leucine and	3	586 219 3028	C=44;O=3;E=(
TGF-beta signaling	4	4609 1875 7057	C=84;O=4;E=(
N-Glycan biosynthe	3	29929 91869 84	C=49;O=3;E=(
RNA transport	5	10940 50628 10	C=151;O=5;E=
Pyrimidine metabol	4	84172 4860 555	C=99;O=4;E=(
Arginine and prolin	3	4128 1152 219	C=54;O=3;E=(

KLF response eleme	360		
**PathwayName**	**#Gene**	**EntrezGene**	**Statistics**
Axon guidance	13	8633 285220 19	C=129;O=13;
Focal adhesion	10	3725 10398 390	C=200;O=10;
ErbB signaling path	6	3725 27 2064 52	C=87;O=6;E=
Sphingolipid metab	4	55304 340485 4	C=40;O=4;E=
Adherens junction	5	1387 2064 5117	C=73;O=5;E=
Renal cell carcinom	5	3725 1387 4233	C=70;O=5;E=
Glycosphingolipid b	3	6489 6482 3081	C=15;O=3;E=
Pathways in cancer	10	650 3725 1387 2	C=326;O=10;
Protein digestion ar	5	1803 1290 2343	C=81;O=5;E=
Circadian rhythm -	3	6096 8945 406	C=22;O=3;E=
Regulation of actin	7	3678 10398 293	C=213;O=7;E
Mucin type O-Glyca	3	51301 55568 64	C=30;O=3;E=
p53 signaling pathw	4	7161 900 27244	C=68;O=4;E=
Bacterial invasion o	4	3678 4233 5291	C=70;O=4;E=
Metabolism of xenc	4	2952 2941 7365	C=71;O=4;E=
Drug metabolism -	4	2952 2941 7365	C=73;O=4;E=
Arrhythmogenic rig	4	3678 51176 100	C=74;O=4;E=
Cell adhesion molec	5	9076 1000 2594	C=133;O=5;E
Tight junction	5	9414 9076 5520	C=132;O=5;E
Glycosphingolipid b	2	6489 6482	C=14;O=2;E=
ECM-receptor inter	4	3678 3909 1290	C=85;O=4;E=
Carbohydrate diges	3	5291 477 6476	C=44;O=3;E=
Prostate cancer	4	1387 2064 5117	C=89;O=4;E=
Glutathione metabc	3	2952 2941 2939	C=50;O=3;E=
Wnt signaling pathv	5	3725 1387 5117	C=150;O=5;E
Endometrial cancer	3	2064 51176 529	C=52;O=3;E=
Basal cell carcinom	3	650 2737 51176	C=55;O=3;E=
Hedgehog signaling	3	650 2737 8945	C=56;O=3;E=
Acute myeloid leuk	3	51176 6774 529	C=57;O=3;E=
Colorectal cancer	3	3725 51176 529	C=62;O=3;E=
Leukocyte transend	4	9076 10398 529	C=116;O=4;E
Lysosome	4	2799 9583 4758	C=121;O=4;E
Pancreatic cancer	3	2064 6774 5291	C=70;O=3;E=
Neurotrophin signa	4	3725 7161 5291	C=127;O=4;E

KLF start	KLF stop	KLF Sequ	ARE (	ARE start	ARE stop	ARE Seque	ARE Tier	Annotation	Distanc	Gene symb	Reg
842132	842142	GAGCCCA	chr1	842003	842017	TGAGCAAC	Tier Four	Intergenic	12680	LOC10013C	0
904521	904531	GTGGGA(	chr1	904669	904683	AGACCAGC	Tier Four	intron (NM	2649	PLEKHN1	0
1074131	1074141	AGGGCT(	chr1	1074142	1074156	AGCACAGC	Tier Four	intron (NR_	1739	RP11-465B	0
1193789	1193799	AGCCCCA	chr1	1193863	1193877	AGAGCACC	Tier three	intron (NM	-11692	FAM132A	0
1282318	1282328	AGCCCCT	chr1	1282346	1282360	AGCACTGT	Tier three	intron (NM	2169	DVL1	0
1293519	1293529	ACCCACT	chr1	1293484	1293498	AGACCCCA	Tier Four	intron (NM	403	MXRA8	1
1396367	1396377	GGACACA	chr1	1396506	1396520	CCACCAGG	Tier Four	intron (NM	-10792	ATAD3B	0
1397831	1397841	GGGGGC(	chr1	1397681	1397695	AGAGCCCT	Tier Four	intron (NM	-9328	ATAD3B	0
1478719	1478729	TGAGGT(	chr1	1478698	1478712	AGAGCTGC	Tier Four	intron (NM	-2984	TMEM240	0
1479590	1479600	CTCCCCA	chr1	1479571	1479585	AGACCAGC	Tier three	intron (NM	-3855	TMEM240	0
1554165	1554175	CCTCCCG	chr1	1554018	1554032	AGGACAGC	Tier Four	intron (NM	2925	MIB2	0
1610881	1610891	GTCCCCA	chr1	1610740	1610754	TGGACAGA	Tier three	intron (NM	13357	SLC35E2B	0
1698641	1698651	caccacgc(	chr1	1698656	1698670	AGAACCTC	Tier three	intron (NM	-8565	NADK	0
1747588	1747598	CTCCTCT(	chr1	1747558	1747572	AGCACTTG	Tier Four	intron (NM	-36085	NADK	0
1831405	1831415	GCCCACA	chr1	1831442	1831456	AGAGCAAC	Tier Four	Intergenic	-8854	GNB1	0
1850169	1850179	GTGGGA(	chr1	1850092	1850106	AGGACAGC	Tier Four	intron (NM	566	TMEM52	1
1854790	1854800	TGGGGA(	chr1	1854883	1854897	CGAACATC	Tier three	Intergenic	-4055	TMEM52	1
1892658	1892668	gggggagg	chr1	1892736	1892750	AGCACAGT	Tier Four	intron (NM	-41923	TMEM52	1
1969238	1969248	ctgggtggg	chr1	1969186	1969200	CGAACACT	Tier Four	Intergenic	-12666	PRKCZ	0
1969238	1969248	ctgggtggg	chr1	1969208	1969221	GAGGAGA	Tier Five	Intergenic	-12666	PRKCZ	0
1969238	1969248	ctgggtggg	chr1	1969186	1969200	CGAACACT	Tier Four	Intergenic	-12666	PRKCZ	0
1969238	1969248	ctgggtggg	chr1	1969208	1969221	GAGGAGA	Tier Five	Intergenic	-12666	PRKCZ	0
2169942	2169952	GGGCACT	chr1	2169883	2169897	GGCACAGC	Tier Four	intron (NM	9813	SKI	-1
2360594	2360604	AAGGGT(	chr1	2360673	2360687	AGCCCTGG	Tier Four	Intergenic	-16589	PEX10	0
2385708	2385718	TGCCCCG	chr1	2385631	2385645	GGAACTGC	Tier Four	Intergenic	-22041	PLCH2	0
2492656	2492666	CAGGGA(	chr1	2492513	2492527	AGAGCAGA	Tier three	intron (NM	-4211	LOC10013	0
2515019	2515029	cagggtgtg	chr1	2515100	2515114	AGAGCAGC	Tier Four	Intergenic	-2875	FAM213B	0
2555569	2555579	gcccccacc	chr1	2555710	2555724	GGACCAGA	Tier Four	intron (NM	8907	MMEL1	0
2575273	2575283	TGGGGT(	chr1	2575218	2575232	ACACCAGC	Tier Four	intron (NM	-10797	MMEL1	0
2702667	2702677	TCCCCCA	chr1	2702638	2702652	AGTACAGC	Tier Four	intron (NM	3558	TTC34	0
2702672	2702682	CACCCCA	chr1	2702638	2702652	AGTACAGC	Tier Four	intron (NM	3553	TTC34	0
2744078	2744088	GTTCCCA	chr1	2744217	2744231	AGAACATG	Tier three	Intergenic	-37853	TTC34	0
2766093	2766103	CTGGGT(	chr1	2766017	2766031	AGGACACA	Tier Four	Intergenic	-59868	TTC34	0
2766125	2766135	CTGGGC(	chr1	2766017	2766031	AGGACACA	Tier Four	Intergenic	-59900	TTC34	0
2924533	2924543	aggggtgga	chr1	2924571	2924585	AGGACACA	Tier Four	Intergenic	-13508	ACTRT2	0
2931699	2931709	TCCCACA	chr1	2931588	2931602	AGAACAGC	Tier Four	Intergenic	-6342	ACTRT2	0
3185065	3185075	AGGGGC(	chr1	3184994	3185008	CCAACACC	Tier Four	intron (NM	1E+05	MIR4251	0
3284678	3284688	AGGGGA(	chr1	3284721	3284735	GGACCAGC	Tier Four	intron (NM	-86464	ARHGEF16	0
3372117	3372127	GAGGGA(	chr1	3372200	3372214	GGAACAGT	Tier Four	intron (NM	975	ARHGEF16	0
3477239	3477249	AACCCCT	chr1	3477142	3477156	AGGCCCCC	Tier Four	TTS (NR_03	110	MIR551A	0
3482988	3482998	CAGGGT(	chr1	3483072	3483086	AGACCTGC	Tier Four	intron (NM	-5639	MIR551A	0
3540022	3540032	ctgggcgtg	chr1	3540131	3540145	AGACCACA	Tier Four	Intergenic	-1529	TPRG1L	-1
3634774	3634784	cggggcgg	chr1	3634652	3634666	AGACCTGT	Tier Four	intron (NM	20169	TP73	-1
3634779	3634789	cggggcgg	chr1	3634652	3634666	AGACCTGT	Tier Four	intron (NM	20174	TP73	-1
3690068	3690078	GACCCCT	chr1	3690003	3690017	AGAACACC	Tier two	intron (NM	722	SMIM1	0
3690068	3690078	GACCCCT	chr1	3690052	3690066	TGAACACA	Tier Four	intron (NM	722	SMIM1	0

3690068	3690078	GACCCCT	chr1	3690003	3690017	AGAACAC	Tier two	intron (NM	722	SMIM1	0
3690068	3690078	GACCCCT	chr1	3690052	3690066	TGAACACA	Tier Four	intron (NM	722	SMIM1	0
3700233	3700243	GTGGGT	chr1	3700102	3700116	AGAACAA	Tier two	intron (NM	10887	SMIM1	0
3732013	3732023	CGGGGT	chr1	3731882	3731896	AGAACTCT	Tier Four	exon (NM_	-18950	LRRC47	0
4190211	4190221	AAGGCT	chr1	4190076	4190090	AGAACTCG	Tier Four	Intergenic	2E+05	RP13-614K	0
4256014	4256024	ctgggtgtg	chr1	4255958	4255972	AGACCATC	Tier Four	Intergenic	-2E+05	RP5-1166F	0
4339913	4339923	GGGGGA	chr1	4339829	4339843	TGAACACA	Tier three	Intergenic	-1E+05	RP5-1166F	0
4370787	4370797	AGGAGC	chr1	4370823	4370837	AGAACGG	Tier Four	Intergenic	-1E+05	RP5-1166F	0
4404230	4404240	AGCCCC	chr1	4404204	4404218	AGAACAG	Tier three	Intergenic	-67876	RP5-1166F	0
4524214	4524224	TGCCCCA	chr1	4524239	4524253	TGCACACC	Tier Four	Intergenic	52108	RP5-1166F	0
4524214	4524224	TGCCCCA	chr1	4524113	4524126	CAGGAGA	Tier Five	Intergenic	52108	RP5-1166F	0
4524214	4524224	TGCCCCA	chr1	4524239	4524253	TGCACACC	Tier Four	Intergenic	52108	RP5-1166F	0
4524214	4524224	TGCCCCA	chr1	4524113	4524126	CAGGAGA	Tier Five	Intergenic	52108	RP5-1166F	0
4613337	4613347	ATCTACA	chr1	4613470	4613484	AGATCATG	Tier three	Intergenic	-1E+05	AJAP1	0
4912674	4912684	AAGCACT	chr1	4912728	4912742	AGAGCAA	Tier Four	Intergenic	2E+05	AJAP1	0
4939600	4939610	TGTCCCT	chr1	4939711	4939725	GGAACAG	Tier Four	Intergenic	2E+05	AJAP1	0
5161825	5161834	atgggggc	chr1	5161844	5161857	CACCAGAA	Tier Five	Intergenic	4E+05	AJAP1	0
5202924	5202934	GAGGGA	chr1	5203016	5203030	AGGGCAA	Tier Four	Intergenic	-4E+05	MIR4417	0
5277538	5277548	GTCTCCA	chr1	5277465	5277479	AGTCCACA	Tier three	Intergenic	-3E+05	MIR4417	0
5295720	5295730	CTCTCCA	chr1	5295823	5295837	AGAACTTC	Tier three	Intergenic	-3E+05	MIR4417	0
5664799	5664809	aagcacac	chr1	5664913	5664927	AGAACACT	Tier three	Intergenic	40673	MIR4417	0
5731289	5731299	GAGGGA	chr1	5731316	5731330	AGAACATC	Tier Four	Intergenic	1E+05	MIR4417	0
5802286	5802296	CGCCCCA	chr1	5802167	5802181	GGAACAA	Tier Four	Intergenic	1E+05	MIR4689	0
5803782	5803792	CTACCCA	chr1	5803641	5803655	AGAGCTTC	Tier three	Intergenic	1E+05	MIR4689	0
5830417	5830427	gtgggtgg	chr1	5830447	5830461	TGGCCAG	Tier Four	Intergenic	92379	MIR4689	0
5875348	5875358	TAGGGT	chr1	5875436	5875450	GGAACAA	Tier three	Intergenic	47448	MIR4689	0
5952037	5952047	GGGGGT	chr1	5951971	5951985	AGCACAAT	Tier Four	intron (NM	-29241	MIR4689	0
6074955	6074965	AGGGCT	chr1	6075001	6075015	AGGACAG	Tier three	intron (NM	-11113	KCNAB2	0
6319759	6319769	CAGGGC	chr1	6319812	6319826	GGGACACT	Tier Four	intron (NM	1271	GPR153	0
6869521	6869531	aagggagg	chr1	6869463	6869477	AGATCAG	Tier Four	intron (NR_	24142	CAMTA1	0
6876631	6876641	GGGGGA	chr1	6876525	6876539	AGAACACA	Tier three	intron (NR_	31252	CAMTA1	0
6975529	6975539	CAGGCT	chr1	6975642	6975656	TGGACAAC	Tier Four	intron (NM	1E+05	CAMTA1	0
6979233	6979243	AGGGAC	chr1	6979118	6979132	AAAACACT	Tier three	intron (NM	1E+05	CAMTA1	0
7003432	7003442	AGGGGA	chr1	7003466	7003480	AGTGCAAC	Tier Four	intron (NM	2E+05	CAMTA1	0
7003498	7003508	CGCCCCA	chr1	7003466	7003480	AGTGCAAC	Tier Four	intron (NM	2E+05	CAMTA1	0
7363814	7363824	tgggacgtg	chr1	7363667	7363681	AGAACCCA	Tier two	intron (NM	-5E+05	VAMP3	-1
7665880	7665890	ggctccacc	chr1	7665990	7666004	AGAGCACT	Tier three	intron (NM	-2E+05	VAMP3	-1
7675372	7675382	AGTCCCT	chr1	7675273	7675287	AGCACTGT	Tier Four	intron (NM	-2E+05	VAMP3	-1
7718482	7718492	ggcaccac	chr1	7718415	7718429	AGAACAC	Tier three	intron (NM	-1E+05	VAMP3	-1
8152802	8152812	AAGGGT	chr1	8152935	8152949	TGAACAG	Tier Four	Intergenic	-66414	ERRFI1	0
8302013	8302023	AGGGGT	chr1	8301939	8301953	AGGTCATG	Tier Four	Intergenic	-82372	SLC45A1	0
8326248	8326258	caccacgc	chr1	8326166	8326180	AGACCAG	Tier Four	Intergenic	-58137	SLC45A1	0
8508176	8508186	TGAGGT	chr1	8508161	8508175	AGAAGAT	Tier two	intron (NM	-24434	RERE	0
8508176	8508186	TGAGGT	chr1	8508125	8508138	GCCCAGA	Tier Five	intron (NM	-24434	RERE	0
8508176	8508186	TGAGGT	chr1	8508161	8508175	AGAAGAT	Tier two	intron (NM	-24434	RERE	0
8508176	8508186	TGAGGT	chr1	8508125	8508138	GCCCAGA	Tier Five	intron (NM	-24434	RERE	0
8514586	8514596	ctgggtgtg	chr1	8514460	8514474	AGACCAA	Tier Four	intron (NM	-30844	RERE	0

Refinement of the androgen response element based on ChIP-Seq in androgen-insensitive and androgen-responsive prostate cancer cell lines

Stephen Wilson [1], Jianfei Qi [2], Fabian V. Filipp [1]

[1] Systems Biology and Cancer Metabolism, Program for Quantitative Systems Biology, University of California Merced, 2500 North Lake Road, Merced, CA 95343, USA; [2] Marlene and Stewart Greenebaum Cancer Center, Department of Biochemistry and Molecular Biology, University of Maryland School of Medicine, 655 West Baltimore Street, Baltimore MD 21201, USA.

Address: 2500 North Lake Road, Merced, CA 95343, USA

Phone: +1-858-349-0349

e-mail: filipp@ucmerced.edu

Supplementary figure 1: A) Western blot and B) qPCR of AR shRNA knockdown.

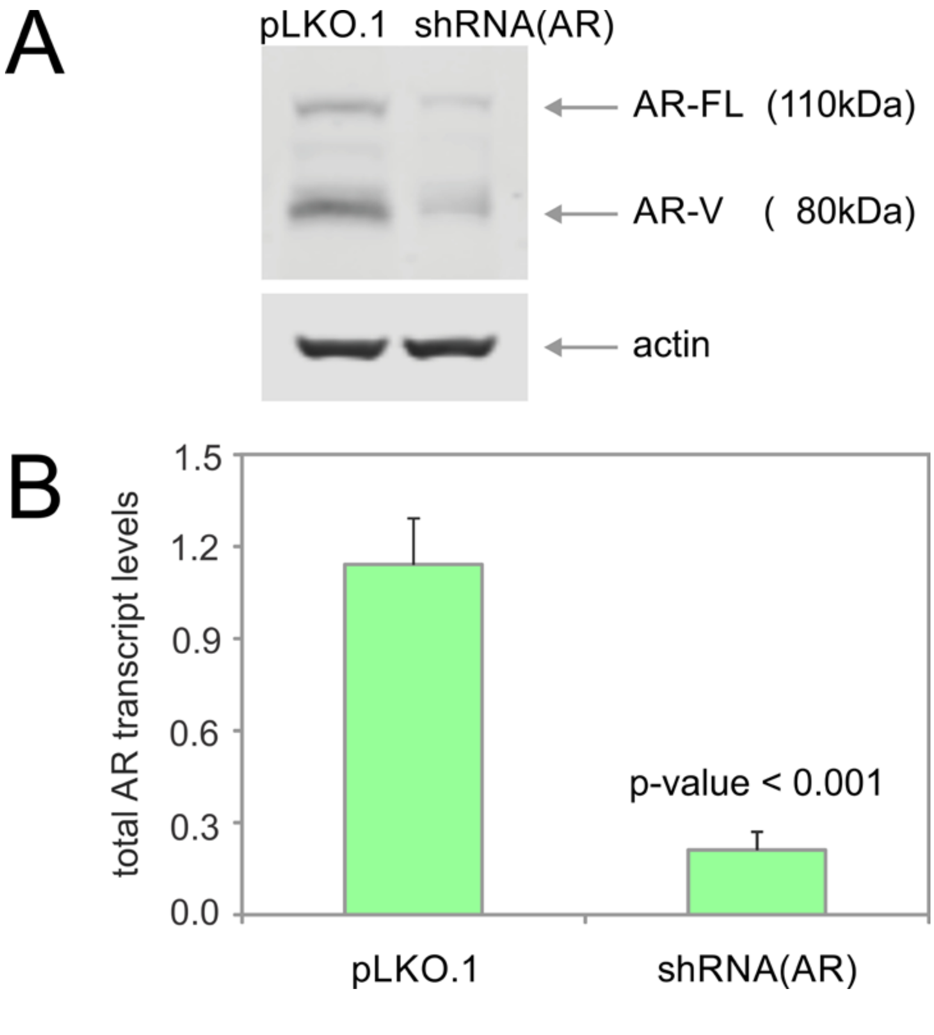

Supplement: Supplementary Information [file srep32611-s2.pdf]
